# Impacts of Atmospheric and Soil Droughts on Carbon and Water Fluxes in Northern European Coniferous Forests: Evidence From Eddy‐Covariance Data

**DOI:** 10.1111/gcb.70962

**Published:** 2026-08-05

**Authors:** Olli Peltola, Olli‐Pekka Tikkasalo, Jari‐Pekka Nousu, Janne Rinne, Samuli Launiainen

**Affiliations:** ^1^ Natural Resources Institute Finland Helsinki Finland

**Keywords:** atmospheric drought, carbon flux, coniferous forest, eddy covariance, extreme conditions, forest growth, soil drought

## Abstract

The recent decline in forest carbon (C) sink across the Nordic countries has been partly attributed to increased water limitations, that is, droughts, on forest productivity. However, empirical evidence for this remains limited. This study combines ERA5‐Land reanalysis datasets with long‐term C and water flux observations from Swedish and Finnish coniferous forest eddy covariance (EC) towers to assess the conditions under which atmospheric (high vapor pressure deficit, VPD) and soil droughts (low soil moisture) have affected forest carbon uptake. Using the reanalysis datasets, we then evaluate whether such conditions have become more common at the studied sites, and across northern Europe in general. EC data suggests that C and water exchange has responded only to extremely low soil moisture, whereas the response to atmospheric droughts has been more gradual and the strongest response occurred during compound droughts. At two sites, isolated drought years led to over 10% reduction in annual gross primary productivity and 20%–40% decline in annual net ecosystem productivity. However, frequent and widespread impacts on annual C fluxes were not observed. The occurrence of atmospheric droughts has increased across northern Europe during the last decade, while soil and compound droughts showed more localized changes. Beyond methodological advances in analyzing drought signals from EC data, our results shed light on how droughts may have influenced ecosystem–atmosphere carbon and water fluxes across northern European forests by integrating site‐level flux responses with reanalysis datasets. The results suggest that increasing VPD may be one underlying factor for the observed decline in forest growth in the Nordic countries, while soil moisture limitations have been likely rarer and more local.

## Graphical abstract

Atmospheric and soil droughts were assessed using the SAMI and SSMI indices at northern European coniferous forest eddy‐covariance (EC) sites, and ecosystem responses were evaluated from EC flux data. The results show that soil droughts have been rare, while air moisture limitations on carbon and water fluxes occur more frequently. Atmospheric droughts have increased across northern Europe over the past decade, whereas soil droughts exhibit more localized trends. The graphical abstract illustrates the distribution of growing‐season days during the 2018 drought (blue contours) and the 1950–2020 reference period (green areas) at an EC site in southern Sweden (SE‐Htm).

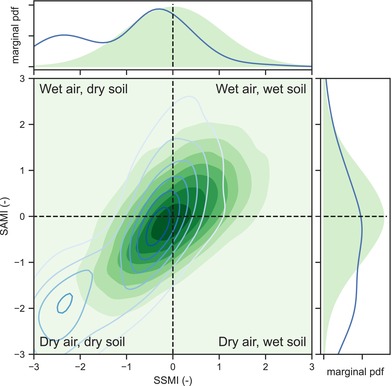

## 1 Introduction

National greenhouse gas inventories indicate that forest carbon (C) sink is decreasing in the Europe and already challenging the forest and climate policies (European Environment Agency 2026; Migliavacca et al. 2025; Hyyrynen et al. 2023; Korosuo et al. 2023). In the Nordic countries, decrease of forest land C sink has been attributed to increasing harvests and natural mortality, and weakening soil C sink, but also to reduced forest growth rate (Henttonen et al. 2024; Laudon et al. 2024; Hyyrynen et al. 2023). Whether the decline in forest productivity is mainly due to forest aging and altered management or warming and drying climate remains unknown, and the drivers can also vary across regions and species (Henttonen et al. 2024; Merlin et al. 2024; Mäkinen et al. 2022; Mensah et al. 2023). Laudon et al. (2024) argued the growth decline of northern forests in Europe should serve as a wake‐up call to improve mechanistic insights on the novel growth‐regulating factors, resulting from direct (past management) and indirect (climate change) human impacts. They hypothesize that increasing water limitations can underlie the growth decline, supported by Mirabel et al. (2023). However, the evidence of climate‐driven‐ decline in forest growth and C sink in the Northern Europe remains anecdotal (Mäkinen et al. 2022; Aldea et al. 2024; van der Woude et al. 2023; Lindroth et al. 2020), and, for instance, in Norway, soil water limitations have so far been considered insignificant (Merlin et al. 2024).

Repeated forest inventories and tree core data enable assessing the diameter or volume growth variability on inter‐annual timescales (Henttonen et al. 2024; Merlin et al. 2024), but do not offer direct insight to physiological mechanisms that regulate the growth and tree C sink variability. Therefore, the hypothesis that increasing water limitations are starting to restrict forest productivity and C sink must be explored by other methods such as long‐term C and water flux measurements (Lindroth et al. 2020; Launiainen, Katul, et al. 2022) or tree‐ring stable isotope records of C and oxygen (McCarroll and Loader 2004; Tang et al. 2023). Eddy‐covariance (EC) data from ICOS (Integrated Carbon Observation System; Heiskanen et al. 2022) and FluxNet (Baldocchi 2020) can reveal immediate, often short‐term, effects of anomalous and extreme environmental conditions on net ecosystem productivity (NEP), gross‐primary productivity (GPP) and forest water use (e.g., Shekhar et al. 2023; Pohl, Werban, et al. 2023; Novick et al. 2016; von Buttlar et al. 2018; Lindroth et al. 2020). The EC data has, however, rarely been used to assess the impacts of drought on C and water fluxes in the Nordic region (Lindroth et al. 2020). Therefore, it remains unknown how frequent physiological droughts have been in Northern European forests, and how large effect the drought‐induced flux anomalies can have on annual NEP, GPP and ecosystem respiration (Reco).

Direct impacts of high vapor pressure deficit (VPD, atmospheric drought) and limited root zone water availability (soil drought) on plant functions and C uptake are relatively well known (e.g., Hao et al. 2025; Wolf and Paul‐Limoges 2023). High VPD induces stomatal closure, directly limiting CO_2_ diffusion from the atmosphere to leaf and constraining leaf and ecosystem level photosynthesis (Novick et al. 2016, 2024; Grossiord et al. 2020). At an ecosystem level, high VPD reduces surface conductance (G_S_), which affects latent heat flux (LE, i.e., evapotranspiration ET ~ G_S_ × VPD) and evaporative fraction (EF = LE/(LE + H), where H is the sensible heat flux). GPP and light‐use efficiency are often less affected by droughts than ET and energy partitioning (Novick et al. 2024). However, when high VPD is associated with limited soil water availability, decreased ecosystem photosynthesis and light‐use efficiency is common (Grossiord et al. 2020; Novick et al. 2024).

Soil drought develops gradually due to the imbalance between soil water inputs (infiltration) and outputs (plant transpiration, soil evaporation, and drainage). During progressive soil drought, soil and leaf water potential gradually decrease and the role of stomatal control of gas‐exchange intensifies (Grossiord et al. 2020; Lin et al. 2015), and non‐stomatal limitations gain in importance as mesophyll conductance and photosynthetic capacity decrease (Keenan et al. 2010; Lin et al. 2015; Grossiord et al. 2020). Soil water availability exhibits a threshold response, characterized by a wide non‐limiting regime, below which dropping soil water potential and reduced soil‐to‐root hydraulic conductivity start to rapidly impact tree hydraulics, water use and GPP (e.g., Sperry et al. 2002; Wood et al. 2023; Fu, Ciais, Prentice, et al. 2022). Compared to GPP and energy partitioning, Reco seems to drop more gradually (Shi et al. 2014), and in Nordic forests, the response of NEP to soil drought is often weaker than that of its components (Lindroth et al. 2020; Launiainen et al. 2015).

Prior research on drought responses of northern European forests C and water cycle using EC observations has provided a blurred image. For instance, the synthesis study of Lindroth et al. (2020) showed that annual NEP can either increase or decrease due to droughts, a finding that was partially explained by highly site‐specific responses of the component fluxes, GPP and Reco. However, large fraction of the variability remained unexplained, possibly due to the coarse temporal resolution (annual) of the study. Similarly, Krasnova et al. (2022) found varied drought responses at hemiboreal forests in Estonia. Gao et al. (2017) showed that in a mixed conifer forest in southern Finland, soil drought affected ecosystem water use efficiency on only a handful of days during a 11‐year study period. Likewise, Matkala et al. (2021) did not observe strong ecosystem responses to extreme weather events at two coniferous forests in Northern Finland. Lately, Scapucci et al. (2024) and Shekhar, Hörtnagl, et al. (2024) have highlighted the importance of compound droughts, that is, joint atmospheric and soil drought, on C and water fluxes at a temperate forest. Previous studies on drought responses of northern European forest C and water fluxes have used varied drought metrics and lack a unified definition of physiological drought. Also, timescales of interest and approaches to disentangle the drought responses and separate them from all other sources of variability in ecosystem scale fluxes lack consistence and hinder direct comparisons between individual studies.

Here, we quantify the effect of high atmospheric VPD, often associated with elevated temperatures, limited soil water availability and their combined effect on forest NEP, its components and EF using long‐term ecosystem scale C, water and energy flux data from seven boreal and hemiboreal coniferous forests located between 56° and 68° N in Sweden and Finland. We formulate non‐dimensional air and soil moisture indices based on site‐specific long‐term VPD and soil moisture statistics and analyze spatiotemporal drought patterns in the Nordic‐Baltic region using ERA5‐Land data (Muñoz‐Sabater et al. 2021). At each EC site, we relate the observed daily flux anomalies to extreme atmospheric or soil moisture conditions and utilize the found daily responses to evaluate the drought effects on annual scale. Specifically, we address the following questions:

1. Can physiological drought signals be observed in the ecosystem C and water fluxes, and to which degree the anomalies can be attributed to atmospheric or soil droughts?
2. How severe, frequent and persistent are high VPD and soil water limitations on forest NEP, GPP, Reco and evaporative fraction, and what is their impact on annual C fluxes?
3. How severe atmospheric and soil droughts have varied in the Nordic‐Baltic region in the past decades, and have they become more common during the last decade?

We use the insights from the EC data and regional drought statistics to discuss whether water limitations can underlie the recent growth decline in the Nordic forests.

## 2 Materials and Methods

### 2.1 Drought Indices

**FIGURE 1:**
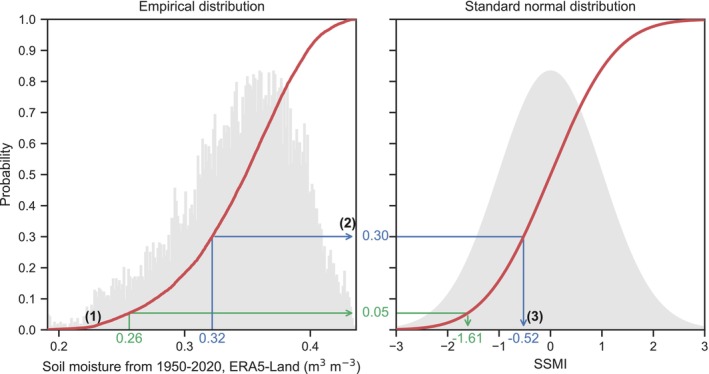
Illustration on calculation of the standardized soil moisture index (SSMI) based on ERA5‐Land data. The volumetric soil moisture content distribution during thermal growing season, at a given grid‐cell, is converted to SSMI using empirical cumulative density function (CDF) and equiprobability transformation. Similar approach was used to convert VPD to SAMI. Red curves show the CDF functions for the empirical distribution (left) and standard normal distribution (right) and grey bars the underlying probability density functions. Estimation of SSMI consists of three steps: (1) estimation of empirical CDF, (2) calculation of probability for each soil moisture value in the time series using the empirical CDF and (3) transformation of this probability into SSMI using inverse of the standard normal CDF. Blue arrows show an example on the calculation procedure where soil moisture value 0.32 m^3^ m^−3^ is converted into SSMI value of −0.52 and green arrows show the conversion between 0.26 m^3^ m^−3^ and −1.61. ERA5‐Land soil moisture data FI‐Hyy area were used in this figure.

We define a standardized soil moisture index (SSMI) and standardized air moisture index (SAMI) to identify and distinguish periods of soil and atmospheric droughts, respectively. These indices were calculated using ERA5‐Land data (Muñoz‐Sabater et al. 2021; Copernicus Climate Change Service 2019), either volumetric soil water content (VWC) between 7 and 28 cm below the soil surface (SSMI) or VPD (SAMI). Data were extracted for the years between 1950 and 2024 for each terrestrial ERA5 grid cell (0.1° × 0.1° horizontal resolution) in the study area (a bounding box spanning from 59°45′ N, 19°15′ E to 70°15′ N, 32°0′ E). Open‐Meteo software (Zippenfenig 2023) was also used in extracting the data. The VPD was calculated from air and dewpoint temperature. SSMI and SAMI were derived based on data from thermal growing seasons only, which were estimated using daily air temperature following Ruosteenoja et al. (2016). Time series of daily SSMI and SAMI were determined separately for each location (EC site or ERA‐5 grid cell) as follows (Figure 1): First, empirical cumulative distribution function (empirical CDF) was calculated from the daily thermal growing season data. Second, the probability of occurrence of each observed soil moisture or VPD value was derived from the empirical CDF. Finally, these probabilities were converted to standard scores using inverse of the standard normal CDF, which yielded the values for the indices. Note that as VPD and temperature are highly correlated, SAMI should be interpreted as a compound index for high VPD and temperature.

**FIGURE 2:**
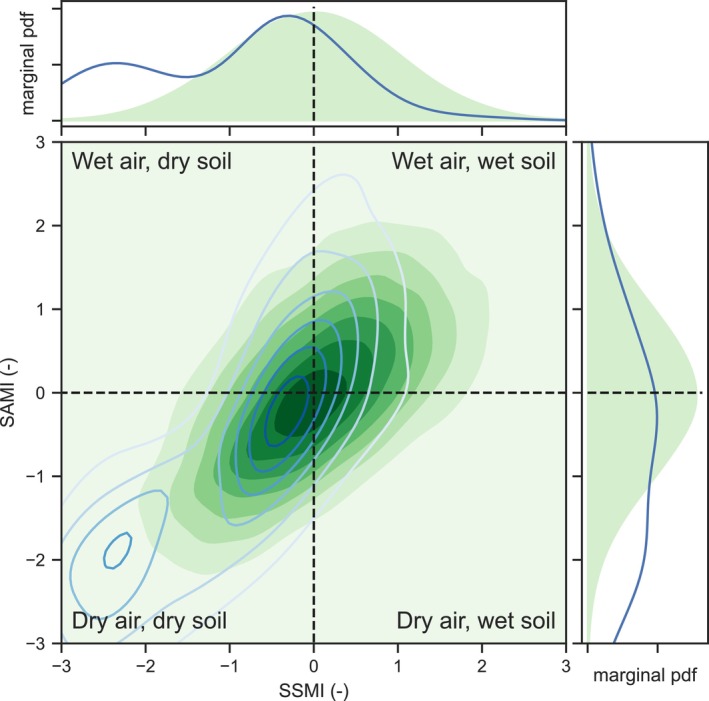
Relationship between soil moisture (SSMI) and air moisture (SAMI) indices at SE‐Htm. The green filled contours show the distribution of growing season data between 1950 and 2020. Blue contours show the same distribution for the European drought Year 2018. SAMI and SSMI values delineate the data into four quadrants: Dry air − dry soil, dry air − wet soil, wet air − wet soil and wet air − dry soil. Marginal probability density functions (marginal pdfs) are shown in the subplots on top and right.

**FIGURE 3:**
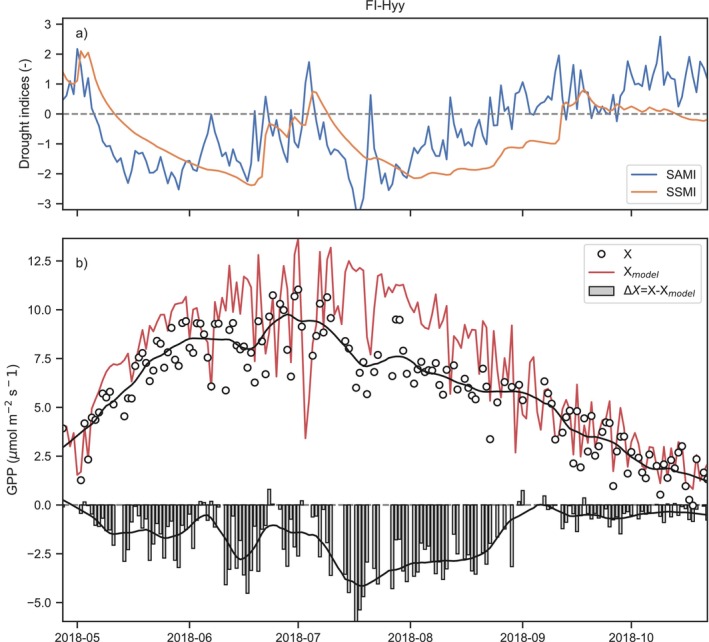
Daily drought indices SAMI and SSMI (a) and measured (*X*) and predicted (*X*_model_) daily mean GPP at FI‐Hyy during the thermal growing season in 2018 (b). Negative SSMI and SAMI indicate that soil and air were drier than normally, respectively. ΔGPP (ΔX in the figure) was calculated as the difference between the predicted (*X*_model_) and measured GPP (*X*), see Equation (6). The continuous black lines (b) show locally estimated scatterplot smoothing (loess) fits to the measured GPP and ΔX.

This approach to produce standardised indices from empirical CDFs is akin to Carrão et al. (2016). Negative SSMI values indicate that the observed VWC was smaller than the most common thermal growing season soil moisture value at that location during the 1950–2020 period, whereas positive SSMI values indicate it was larger. To have the same sign convention for SSMI and SAMI, the SAMI values were multiplied by −1; hence negative SAMI values indicate that VPD was larger than the most common value occurring in the thermal growing season. Mathematically, this procedure to estimate the indices can be written as

(1) $$\text{SAMI}=-F_{n}^{-1}\left(F_{\mathrm{VPD}}\left(\mathrm{VPD}\right)\right)$$

(2) $$\text{SSMI}=F_{n}^{-1}\left(F_{\mathrm{VWC}}\left(\mathrm{VWC}\right)\right),$$

where $F_{n}^{-1}$ is the inverse of the standard normal CDF, $F_{\mathrm{VPD}}$ and $F_{\mathrm{VWC}}$ are the local empirical CDFs for VPD and VWC, respectively, and VPD and VWC are the local time series. Highly negative SSMI (SAMI) indicate that the soil (air) was very dry when compared to the most common conditions at the location. SSMI and SAMI values delineate days into four quadrants representing different combinations of daily soil and air moisture status (Figure 2). Soil moisture variability is typically delayed from the corresponding variability in VPD (Figures 2 and 3a). Figure 3a shows an example of SSMI and SAMI time series at one EC site during one thermal growing season. Some prior studies have used percentiles of soil moisture and VPD time series to identify air and soil droughts. For instance, the 10th and 90th percentiles for soil moisture and VPD used in Shekhar, Buchmann, et al. (2024); Shekhar, Hörtnagl, et al. (2024) translate to SSMI and SAMI values of −1.28. Different approaches for categorising drought severity have been used in the past (Pohl, Rakovec, et al. 2023) and we define index values below −2 as extreme, between −1.5 and −2 as severe, between −1 and −1.5 as moderate and −0.5 and −1 as mild drought. This follows McKee et al. (1993) with the exception for the definition of mild drought. Note that the indices SAMI and SSMI do not explicitly consider the drought persistence in their definitions. However extremely low SSMI values are acquired only when the ecosystem water balance has been negative over an extended period of time and hence the drought persistence is implicitly included in the metric definition. Furthermore, the study results were not sensitive to the length of the past soil drought periods (see Figures S12 and S13 and the corresponding text in the Supporting Information) suggesting that the observed ecosystem responses were largely in synchrony with the drought metric variability.

Our definition of drought indices is akin to Carrão et al. (2016) and Pohl, Rakovec, et al. (2023) who estimated SSMI from soil moisture data; however, there are few important differences: (1) our analyses focus exclusively on the physiologically relevant thermal growing season, (2) we utilize empirical CDFs, and (3) our indices are based on ERA5‐Land data. Also, they did not analyze atmospheric droughts via SAMI or compound droughts with both SAMI and SSMI. The use of empirical CDFs instead of parametric distributions eliminates the need to assume a certain functional form for the underlying PDF or go through uncertain bandwidth selection in kernel density estimation (KDE, Pohl, Rakovec, et al. 2023). Furthermore, VPD and SWC are bounded random variables, and KDE tends to produce uncertain values near the bounds (Carrão et al. 2016), while fitting parametric distributions is uncertain in the distribution tails (Farahmand and AghaKouchak 2015). No detrending or deseasonalizing was done to the VPD or VWC time series before calculating the indices. See the Supporting Information for a comparison against indices calculated from deseasonalized data.

### 2.2 Eddy Covariance Observations and the Metrics of Ecosystem Function

EC flux data from seven ICOS forest ecosystem sites in Finland and Sweden were used in this study, covering the climate gradient from hemiboreal southern Sweden to boreal/sub‐arctic Northern Finland (Table 1). All sites are either Scots pine ( *Pinus sylvestris* ), Norway spruce ( *Picea abies* ) or mixed evergreen coniferous forests with mean stand age from 35 to 122 years Data from FI‐Ken, SE‐Nor, SE‐Htm, SE‐Svb and SE‐Ros sites were retrieved from ICOS carbon portal, and data processing was executed by ICOS (Sabbatini et al. 2018; Vitale et al. 2020; Pastorello et al. 2020). To utilize the long time series measured prior to ICOS, we retrieved locally processed data for FI‐Var and FI‐Hyy from the site PI's (Mammarella et al. 2016; Launiainen, Kieloaho, et al. 2022). The flux time series were gapfilled with the marginal distribution sampling algorithm, and NEP measured by EC was partitioned into GPP and Reco (NEP = GPP − Reco) with the nighttime partitioning method (Reichstein et al. 2005; Pastorello et al. 2020). For FI‐Hyy and FI‐Var these two processing steps were executed using the REddyProc tool that use routines that are similar to the ICOS protocols (Wutzler et al. 2018). Days for which less than one third of the data were measured were excluded from further analysis.

**TABLE 1:** Description of the EC measurement stations included in this study. Stand age refers to stand age in 2018.

Site‐id	Site	Lat, lon	Tree species	LAI (m^2^ m^−2^)	Stand age (year)	Site type	Soil type	Habitat	Site years	Data source	References
FI‐Hyy	Hyytiälä	61°51′ N, 24°18′ E	Scots pine (60%), Norway spruce (25%), Birch (15%)	3.2–4.5	56	Sub‐xeric	Fine till	Boreal	1997–2019	Launiainen, Katul, et al. (2022)	Launiainen, Kieloaho, et al. (2022)
FI‐Ken	Kenttärova	67°59′ N, 24°15′ E	Norway spruce	2.8	98	Sub‐xeric	Fine till	Boreal	2018–2023	Laurila et al. (2024)	Aurela et al. (2015)
FI‐Var	Värriö	67°45′ N, 29°37′ E	Scots Pine	1.6	68	Xeric	Sandy till	Boreal	2013–2023	Kolari et al. (2023)	Kulmala et al. (2019)
SE‐Nor	Norunda	60°05′ N, 17°29′ E	Scots pine (62%), Norway spruce (37%), Birch (1%)	4.4	122	Mesic	Sandy till	Hemiboreal	2014–2020	Mölder et al. (2022)	Lindroth et al. (2018)
SE‐Htm	Hyltemossa	56°06′ N, 13°25′ E	Norway Spruce	5.7	35	Mesic	Sandy till	Hemiboreal	2015–2020	Heliasz et al. (2022)	Lindroth et al. (2020)
SE‐Svb	Svartberget	64°15′ N, 19°46′ E	Scots pine (61%), Norway spruce (34%), Birch (5%)	4.6	95	Sub‐xeric	Fine till	Boreal	2014–2020	Peichl et al. (2022)	Chi et al. (2020)
SE‐Ros	Rosinedal	64°10′ N, 19°45′ E	Scots pine	2.7	95	Sub‐xeric	Fine till	Boreal	2014–2020	Peichl et al. (2022)	Chi et al. (2021)

Ecosystem light response was assessed for each day using the rectangular hyperbolic light response curve (Falge et al. 2001):

(3) $$\mathrm{GPP}=\frac{\alpha P_{\max}\text{PPFD}}{\text{αPPFD}+P_{\max}},$$

where α (−) is apparent quantum yield, $P_{\max}$ (μmol m^−2^ s^−1^) is ecosystem photosynthesis without light limitation and PPFD (μmol m^−2^ s^−1^) is photosynthetic photon flux density. Equation (3) was fitted to 30‐min values of GPP and PPFD observed during each day, resulting in daily time series of α and $P_{\max}$. Similarly, temperature response of Reco was assessed following Lloyd and Taylor (1994):

(4) $$\text{Reco}=R_{\mathrm{ref}}e^{E_{0}\left(\frac{1}{T_{\mathrm{ref}}-T_{0}}-\frac{1}{T-T_{0}}\right)},$$

where $E_{0}$ (K^−1^) is the temperature sensitivity of respiration, T (°C) is air temperature, $T_{\mathrm{ref}}=15^{{}^{\circ}}C$, $T_{0}=-46.02^{{}^{\circ}}C$ and $R_{\mathrm{ref}}$ (μmol m^−2^ s^−1^) is the baseline respiration describing Reco when $T=T_{\mathrm{ref}}$. Fixed value for $E_{0}$ was utilized for each site and estimated (Reichstein et al. 2005), whereas $R_{\mathrm{ref}}$ was estimated separately for each day using Equation (4), similarly as in the case of GPP. Note that as we utilize data from nighttime partitioning method, the Reco temperature response is estimated from already modelled data. These analyses provided daily values for the parameters describing GPP light response and Reco temperature response that were used in estimating the drought responses (see below) The evaporative fraction (EF) was calculated as

(5) $$\mathrm{EF}=\frac{\mathrm{LE}}{\mathrm{LE}+H},$$

where LE and H are daily latent and sensible heat fluxes, respectively. EF quantifies the fraction of available energy used in evapotranspiration and hence it likely varies in response to moisture availability (e.g., Seneviratne et al. 2010).

Atmospheric and soil droughts are expected to alter ecosystem functions, resulting in anomalies in ecosystem C fluxes, GPP and Reco response curves (Equations (3) and (4)) and EF. We analyzed the anomalies ΔX in daily time series as follows:

(6) $$\mathrm{\Delta X}=X–X_{\text{model}},$$

where *X* is the observed daily time series and *X*_model_ is a statistical model (see below) describing the expected value for X at each time step without VPD or soil moisture limitations. Ideally, ΔX describes the ecosystem response to moisture stress. However, due to our definition of *X*_model_, there can be residual effects outside VPD and soil moisture limitation that are shown in ΔX. These residual effects are later discussed in Section 3.1. For α, $P_{\max}$, $R_{\mathrm{ref}}$ and EF, the *X*_model_ was estimated akin to von Buttlar et al. (2018). It was estimated for each day as the median *X* observed at that particular day of year (DOY); however for FI‐Hyy and FI‐Var it was estimated as a linear fit between *X* and year separately for each DOY due to the long time series available from these sites, albeit measurement setup changes may complicate this analysis at FI‐Hyy (Launiainen, Katul, et al. 2022). Furthermore, when deriving *X*_model_ for a specific year, the data from that particular year were excluded and hence the derived *X*_model_ values were independent of the corresponding observations. For removing stochastic noise from the *X*_model_ time series it was then smoothed with 14 days centered averaging. Then, for GPP and Reco, the *X*_model_ was estimated with Equations (3) and (4), respectively, by utilizing the modelled values for the fit parameters obtained in the previous step and, for NEP, the *X*_model_ was calculated using the *X*_model_ for GPP and Reco. This way the models for GPP, Reco and NEP capture the flux seasonality, as well as their short‐term responses to their most important environmental drivers, that is, PPFD for GPP, T for Reco and both for NEP. Finally, the anomalies ΔX were normalised to zero mean and unit variance for easier comparison between sites and variables. Figure 3 shows an example of SSMI and SAMI time series along with observed (*X*) and modelled (*X*_model_) GPP at FI‐Hyy and their difference (ΔX) during the thermal growing season during the Year 2018.

### 2.3 The Effect of Drought Anomalies on Annual Carbon Fluxes

**FIGURE 4:**
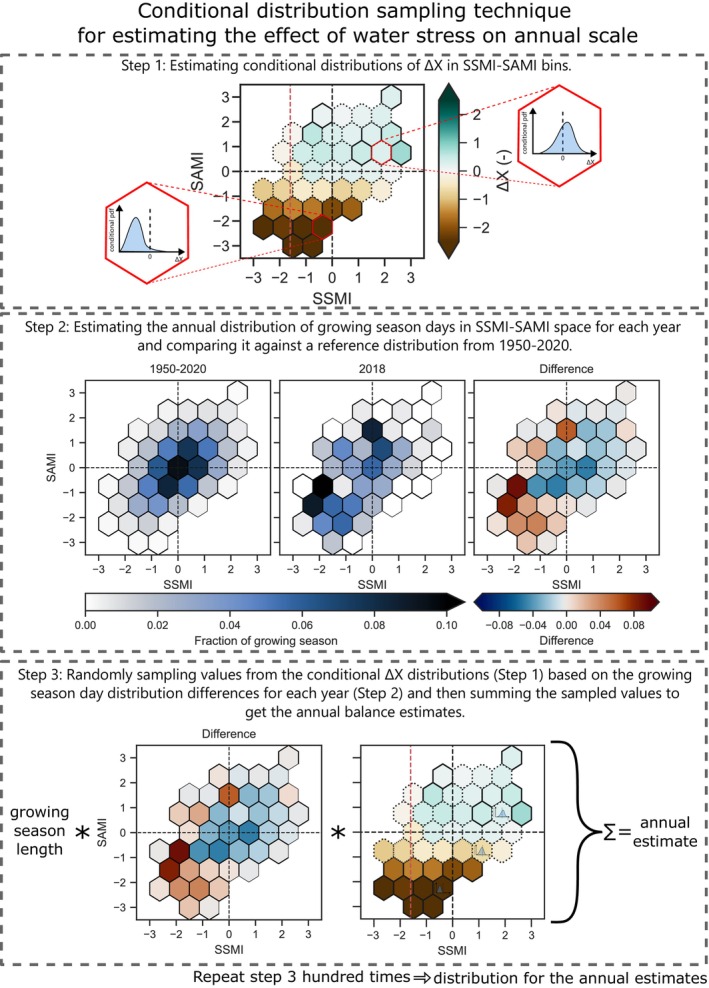
Schematic figure showing how the effect of water stress observed at daily scale was used to evaluate the drought impacts on annual scale. Data from FI‐Hyy were used in this plot.

The effect of atmospheric and soil droughts on annual NEP, GPP and Reco were estimated with a three‐step procedure, see Figure 4. First, the conditional distributions of ΔX values were estimated in SAMI‐SSMI bins. The distributions were estimated with KDE for bins with at least 5 daily data points. Second, fraction of thermal growing season days falling in these SAMI‐SSMI bins were estimated separately for each year and for the full reference period (1950–2020). Then the difference between these fractions were calculated for evaluating how the distributions in a specific year differed from the reference distribution. An example of the distributions in Figure 4 at the FI‐Hyy site show that low SSMI and SAMI values, indicative of soil and atmospheric droughts, were more common during the thermal growing season of 2018 than normally. Third, the over‐ or underrepresentation of each SAMI‐SSMI bin within the thermal growing seasons were considered and ΔX values were randomly sampled from the corresponding SAMI‐SSMI bin specific conditional distributions. Then the sampled ΔX values were summed to produce an estimate for the water stress effect on annual *X* values. For instance, if there were 10 thermal growing season days more in a specific SAMI‐SSMI bin, then 10 ΔX values were independently sampled from the corresponding ΔX conditional distribution. On the other hand, if there were for instance 7 days less in a specific bin, then 7 ΔX values were sampled from the corresponding ΔX conditional distribution and when calculating the sum, these 7 values multiplied with −1 to account for the fact that this bin was underrepresented during this particular year when compared to the reference. This third step was repeated independently 100 times for producing also uncertainty estimates for the annual effects. If the ΔGPP, ΔReco and ΔNEP did not show any clear dependence on SAMI or SSMI, then this procedure yielded an estimate that showed negligible effect of water availability on annual fluxes, whereas strong SAMI and SSMI dependence would result in a strongly altered annual fluxes. For acquiring reasonable estimates for drought effects on annual scale with this procedure, the SAMI‐SSMI bins need to have large enough set of data points for gaining robust estimates for the conditional distributions in each bin and on the other hand there needs to be enough SAMI‐SSMI bins for capturing the ∆X dependence on these two variables. Hence, a balance needs to be struck between these two points of view when deciding the number of bins. Furthermore, the ecosystem response to a particular SAMI‐SSMI state needs to be instantaneous (daily) since in this approach the lagged effects, for example, the effect of the length of the past drought period, are neglected. The analysis included in the Supporting Information (Figures S12 and S13 and the corresponding text) suggests that the past states of the system, that is, length of the past drought period, was not strongly altering the current response to a particular SAMI‐SSMI state, supporting our approach for estimating the drought effects on annual scale from daily scale observations.

### 2.4 Drought Occurrence and Spatial Trends in the Past and During 2015–2024 Period

To analyze the drought occurrence and spatial trends of SSMI and SAMI in the Nordic‐Baltic region, the data was split into reference period (1980–2010) and the most recent decade (2015–2024). For both periods, the mean number of days where thermal growing season SAMI or SSMI was below −2.0 (i.e., extreme conditions) was calculated for each location. For each location, the mean number of days was converted to mean fraction of thermal growing season (TGSD%) by accounting for the inter‐annual variability of thermal growing season length. The anomaly of local TGSD% during the most recent decade was then calculated by subtracting TGSD% of the reference period from TGSD% of the most recent decade.

## 3 Results and Discussion

### 3.1 Flux Anomalies During Atmospheric and Soil Droughts

**FIGURE 5:**
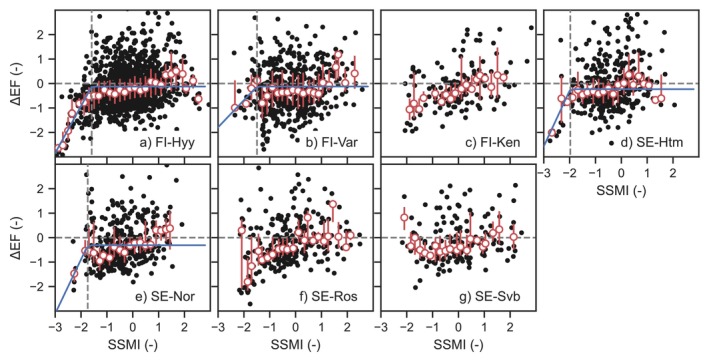
The dependence of standardized evaporative fraction anomaly (ΔEF) on soil moisture index (SSMI) during growing season soil moisture dry‐downs. Black dots show daily values, red circles bin medians (errorbars: Interquartile range) and blue curves the fitted linear‐plus‐plateau models suggested by Fu, Ciais, Feldman, et al. (2022). The fits are shown only if the fit was successful. The vertical dashed lines show the critical SSMI values delineating the soil moisture and energy limited regimes.

Figure 5 shows the clear response of EF on soil water availability (via SSMI) at some, but not all, study sites. Based on a conceptual framework derived from classical hydrology, the transition of the ecosystem water cycle from energy‐limited to water‐limited regime occurs at a critical soil moisture threshold (e.g., Seneviratne et al. 2010). While being a gross simplification of the underlying ecohydrological processes, the framework has recently proven useful in analyzing water stress in global ecosystems (Fu, Ciais, Prentice, et al. 2022; Fu et al. 2024). In the energy‐limited regime, EF does not strongly depend on soil moisture, whereas in the soil moisture‐limited regime, the evapotranspiration rate decreases and EF becomes a quasi‐linear function of soil moisture. We evaluated the dependence of ΔEF on SSMI during periods when soil moisture was decreasing for at least 10 consecutive days (dry‐downs) and identified the two limiting regimes following Fu, Ciais, Prentice, et al. (2022). A clear transition between the regimes was detectable only at FI‐Hyy (mixed conifer, SSMI < −1.6, Figure 5a), FI‐Var (pine, −1.5, Figure 5b), SE‐Htm (spruce, −2.0, Figure 5d), and SE‐Nor (mixed conifer, −1.6, Figure 5e). No change point in the ΔEF versus SSMI relationship was found at the other studied forests, that remained in the energy‐limited regime during the study period. However, note that only a small fraction of the data falls within the dry soil extremes, and these thresholds are thus uncertain (Figure 5).

The findings suggest that at the studied Nordic forests, partitioning of available energy to LE and H has only seldom been affected by soil moisture. Putting the results in perspective, SSMI of −1.6 equals the probability of approx. 0.05 for the ecosystem being in soil moisture limited regime during a thermal growing season. At a site where the average thermal growing season length is 170 days (e.g., FI‐Hyy) this equals ca. 85 thermal growing season days per decade. For SSMI = −2 this would translate to a probability of 0.02 and 34 days. Based on Figure 5, strong soil moisture limitations on EF were observed only when SSMI < −2.5 (FI‐Hyy, SE‐Htm, SE‐Nor); such situations had very low probability (0.006) and hence occurrence (11 days per decade).

**FIGURE 6:**
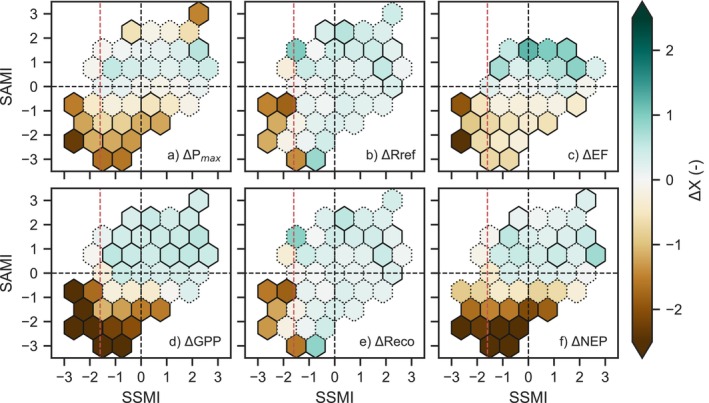
Bin medians of the standardized anomalies of Δ*P*_max_ (a), ΔRref (b), ΔEF (c), ΔGPP (d), ΔReco (e) and ΔNEP (f) in air (SAMI) and soil moisture (SSMI) bins at FI‐Hyy. Only bins with more than 5 data points are shown. Hexagon edges are given with dashed line if 0 is within the interquartile range of the data in the bin. Vertical red dashed line shows the approximate SSMI threshold below which the ecosystem is in soil moisture limited regime (see Section 3.1). When ΔX < 0, the observed values were below the expected values, suggesting; for example, that the ecosystem fluxes (GPP, Reco or NEP) were decreased due to the moisture conditions.

The responses of carbon flux and bulk ecosystem parameter anomalies to SSMI and SAMI at FI‐Hyy site are shown in Figure 6 (see Figures S1–S6 for the other sites). Binning the daily anomalies ΔP_max_, ΔRref, ΔEF, ΔGPP, ΔReco, and ΔNEP against both SSMI and SAMI enables disentangling the ecosystem responses to soil and atmospheric droughts and to evaluate the effect of compound droughts. The response of ΔP_max_ and ΔEF to SAMI was gradual and all the anomalies became negative when SAMI was negative (Figure 6a,c,d), indicating that physiological water stress was increasing gradually with increasing VPD irrespective of soil moisture conditions. Note that as VPD and temperature are strongly correlated, this might partially reflect heat stress as well. Nevertheless, this is in line with earlier studies showing that ecosystems react to increasing atmospheric dryness over a wide range of VPD (Grossiord et al. 2020; Fu, Ciais, Feldman, et al. 2022; Shekhar et al. 2023; Novick et al. 2024). The ΔP_max_ and ΔEF remained insensitive to soil moisture (SSMI) except at the dry extreme below a threshold SSMI value (Figure 6a,c,d). The reference ecosystem respiration rate anomaly (ΔRref) became negative only when SSMI was below approximately −2, but did not have a clear response to SAMI (Figure 6b).

While anomalies in Reco closely follow those of ΔRref, ΔGPP shows a stronger drought response than the light‐saturated photosynthetic rate ΔP_max_. The humid periods with ample soil moisture and low VPD (SSMI & SAMI > 0) are associated with slightly positive ΔGPP, while GPP reduction during compound droughts (SSMI & SAMI < 0) was stronger than that of ΔP_max_ (compare Figure 6a,d). In a relative sense, the response of ΔGPP to soil and atmospheric droughts was stronger than the corresponding response of ΔReco, in accordance with Schwalm et al. (2010). The anomalies in net ecosystem productivity (ΔNEP) follow closely those of ΔGPP, although at moderately negative SAMI the decrease in Reco partly compensates for the dropping GPP (Figure 6d,e). ΔNEP was negative over a wide range of SSMI and SAMI values (Figure 6f), indicative that the ecosystem carbon sink was decreased by both soil and atmospheric droughts but without clear threshold values. The responses of flux anomalies to drought indices were qualitatively similar across the other sites (Figures S1–S6). However, as the study periods and distributions of SAMI and SSMI vary between sites, and their data coverage is shorter compared to FI‐Hyy, this resulted in less clear signals (Figure 6; Figures S1–S6).

Our results show that VPD limitations on ecosystem carbon and water fluxes are more prevalent than soil moisture limitations. VPD provides the primary ecohydrological limitation on ecosystem GPP across a wide range of soil moisture, while soil water availability becomes crucial only in dry soils (Grossiord et al. 2020; Fu, Ciais, Feldman, et al. 2022) during which also the sensitivity to VPD tends to decrease (Novick et al. 2016; Fu, Ciais, Feldman, et al. 2022). Moreover, anomalies in NEP are more related to those in GPP than Reco. Boreal forest stands also show surprisingly rapid recovery of leaf and ecosystem level fluxes after short‐duration droughts (Launiainen et al. 2015; Shekhar et al. 2023). For instance, Shekhar et al. (2023) showed that evergreen needleleaf forest NEP recovered from atmospheric drought on average in 3 days and our analysis on the effect of past soil drought periods on the current ecosystem responses suggest a rapid recovery as well (see Figures S12 and S13 and the corresponding text). The strength of stomatal and non‐stomatal limitations on C and water exchange varies both within forest canopies and across landscapes, as they depend, for example, on tree species, size, position in the canopy, local microclimate and soil hydraulic regime (Niinemets 2010; Grossiord et al. 2020; Novick et al. 2024). Particularly, the sensitivity to VPD tends to differ between isohydric (e.g., Scots pine, Norway spruce) and anisohydric trees, reflecting different safety margins of tree hydraulic system (Grossiord et al. 2020). The effect of species or canopy structure on tree stand drought responses can, however, not be addressed with spatially scarce EC flux data spanning large geographical area.

Our analysis of ΔGPP, and through that ΔNEP residuals, assumes that the underlying models for GPP and Reco (Equation 3 and the X_model_ definition in Section 2.2) capture the dominant variability not related to the soil or atmospheric drought. PPFD is the main driver of photosynthesis and a simple light response function (Equation 3) is commonly used in analysing ecosystem GPP responses to PPFD (e.g., Falge et al. 2001). While the other variables such as fraction of diffuse and direct radiation, solar zenith angle, leaf temperature and nutrient status affect photosynthesis, the available data does not permit us to reliably fit more complex models to estimate ΔGPP.

If the light‐response model systematically failed to capture GPP at the studied sites, we would expect high or low ΔX values also in other quadrants besides low SSMI and SAMI (Figure 6) which are not observed. This supports our hypothesis that ΔGPP and ΔNEP reflect limitations by soil or atmospheric drought. Nevertheless, since temperature is not used for estimating the model for GPP, we note that especially low SAMI values can reflect both high VPD limitation to photosynthesis and leaf heat stress, since air temperature and VPD are tightly correlated.

### 3.2 Impact of Droughts on Annual Carbon Fluxes

**FIGURE 7:**
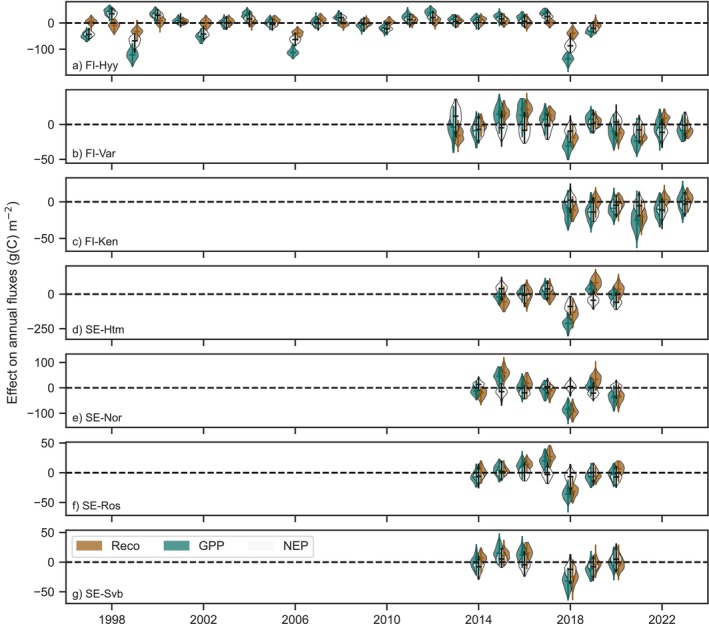
Combined impact of elevated VPD (SAMI) and soil droughts (SSMI) on annual C fluxes at the study sites. Negative values in a given year indicate a decrease of annual NEP, GPP or Reco due to stronger‐than‐average droughts. Horizontal lines show distribution medians. Note the *y*‐axis scale differs between the subplots representing different EC‐sites.

Atmospheric dryness and soil water limitations clearly decreased annual GPP at the FI‐Hyy mixed conifer forest during the years 1997, 1999, 2002, 2006, 2010, 2018 and 2019 (Figure 7a). However, only during 1999, 2006 and 2018, the drought‐induced GPP deficit effect amounted to more than 10% of the corresponding observed annual GPP (Figure 7a). Annual ecosystem respiration was less sensitive to droughts than GPP, meaning the droughts reduced annual NEP. For instance, the NEP decrease of ca. −60 g(C) m^−2^ a^−1^ (−80 … −40 g(C) m^−2^ a^−1^, 95% confidence interval) in 2006 corresponds to ca. one fourth of the long‐term mean NEP (230 g C m^−2^ a^−1^). No strong influence of droughts on annual fluxes were found for the FI‐Var (Scots pine) and FI‐Ken (Norway spruce) sites in northern Finland (Figure 7b,c). The strongest absolute decrease of annual GPP (−240 g(C) m^−2^ a^−1^ or ca. 10% of long‐term mean GPP) occurred during the intense European 2018 drought (e.g., Lindroth et al. 2020) at the southernmost site SE‐Htm (spruce), while the effects of droughts were less evident at SE‐Svb (mixed) and SE‐Ros (pine) in northern Sweden. At SE‐Nor, SE‐Ros and SE‐Svb, annual Reco decreased roughly in tandem with GPP and annual NEP remained unaltered. In the hemiboreal spruce stand SE‐Htm, the effect of drought on Reco was smaller than on GPP, and annual NEP was significantly decreased (−90 g(C) m^−2^ a^−1^ or ca. 50% of long‐term mean NEP) in 2018, resembling the decrease at FI‐Hyy in 2006. These results are to a degree sensitive to the period used in estimating the annual drought impacts and typically somewhat (on the order of 10 g(C) m^−2^ a^−1^) weaker responses were estimated on annual scale if only the peak summer months (June–August) were considered instead of the whole growing season as done here (see Figures S14 and S15). For instance, the effect of drought on annual GPP during the Year 2018 at SE‐Htm was estimated to be −230 g(C) m^−2^ a^−1^ (−280 … −170 g(C) m^−2^ a^−1^) when estimated from the peak summer months. Nevertheless, the salient features of the findings were retained regardless of which period (June–August or whole growing season) were used in estimating the annual effects.

The estimates of drought impact on annual carbon fluxes were acquired using the daily flux dependencies on SSMI and SAMI (see Section 2.3) and thus ignore several confounding processes. Summer droughts are often preceded by abnormally warm springs and early start of the growing season (e.g., Kljun et al. 2007; Wolf et al. 2016; Smith et al. 2020). This is because both can be caused by the same large‐scale persisting weather phenomena (e.g., Rousi et al. 2022) but also since earlier start of growing season tends to increase early summer ET and lead to earlier depletion of soil moisture (e.g., Buermann et al. 2013). Hence, when the fluxes are viewed on annual scale, the early start of the growing season can partially compensate the decrease of annual carbon fluxes that would result from droughts alone. For instance, Kolari et al. (2009) argued that the annual NEP was not strongly affected by the drought at FI‐Hyy during the Year 2006 due to these compensatory mechanisms. Their findings are at odds with our results, where annual NEP was approximated to be decreased by −60 g(C) m^−2^ a^−1^ due to drought. Similarly, the earlier estimates of European 2018 drought impact on forest annual carbon balance are not fully comparable to our findings. For instance, Lindroth et al. (2020) estimated that annual NEP and GPP were decreased by −250 and −190 g(C) m^−2^ a^−1^ and Reco increased by 60 g(C) m^−2^ a^−1^ at SE‐Htm in response to the 2018 drought, whereas our results suggest that the annual NEP, GPP and Reco were decreased by −90 g(C) m^−2^ a^−1^ (−150 … −30 g(C) m^−2^ a^−1^), −240 g(C) m^−2^ a^−1^ (−290 … −180 g(C) m^−2^ a^−1^) and −130 g(C) m^−2^ a^−1^ (−200 … −60 g(C) m^−2^ a^−1^), respectively. Lindroth et al. (2020) analyzed the drought response comparing annual fluxes from drought and reference years, while our analysis is based on daily flux anomalies relationship with the atmospheric and soil drought indices. To quantify ecosystem responses to droughts the latter approach is necessary, as it focuses on time scales where the drought responses can be isolated from other confounding, and possibly compensatory processes affecting the annual fluxes. It should be, however, noted that our conditional distribution sampling technique (Figure 4) to relate flux anomaly to SAMI and SSMI still ignores the effect of timing of drought periods within the growing season by assuming that there is unique data‐driven distribution between flux and drought anomalies (see Sections 2.3 and Supporting Information; Figures S12–S15). Moreover, our approach does not account for possible legacy effects from previous year(s) (Kannenberg et al. 2020; Pohl, Werban, et al. 2023; Yu et al. 2022).

### 3.3 Occurrence of Soil and Atmospheric Droughts

#### 3.3.1 Occurrence Across Study Sites

**FIGURE 8:**
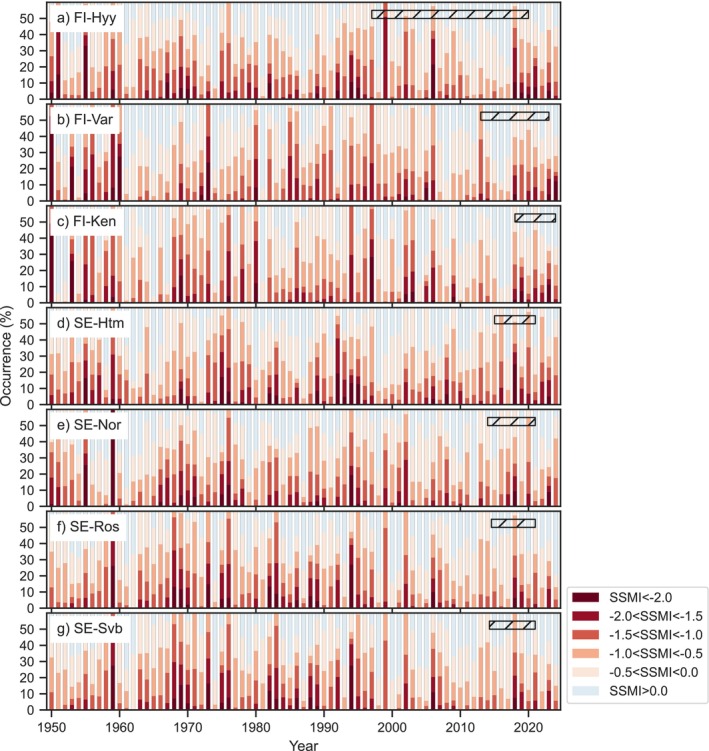
Annual occurrence of different soil moisture index (SSMI) values since the Year 1950. Note that the *y*‐axis is cut at 60% to highlight the occurrence of drought conditions. The more negative the SSMI, the drier the soil. Hatched areas show the temporal coverage of EC data used in this work.

The results indicate that drought indices based on long‐term VPD (SAMI) and ERA5‐Land soil moisture (SSMI) distribution at a given location can be used to identify periods of physiological droughts on Nordic forests NEP and water and energy exchange (Figures 5, 6 and 6). The flux anomalies can be interpreted as effects of elevated VPD and temperature, limited soil water availability or combined drought events (Figure 6), and they can significantly decrease annual NEP during the most extreme years (Figure 7). At the study sites, the occurrence of extreme soil droughts (SSMI < −2), however, has not clearly increased during the recent decade and the variability between the years is prominent (Figure 8). Only the Years 2018 and 2023 stand out from the previous decade during which extreme soil droughts occurred over 10 thermal growing season days at multiple sites (e.g., 57 days during 2018 at SE‐Htm). Severe soil droughts (−1.5 < SSMI < −2) have been more common at all the sites. Extreme atmospheric droughts (SAMI < −2) have occurred frequently during the previous decade, and the inter‐annual variability of SAMI distribution is smaller than SSMI variability at all sites (compare Figures 8 and S8). Typically, only few thermal growing season days per year relate to extreme atmospheric droughts and even less to extremely low soil moisture, limiting the amount of EC data in those bins. The strongest ecosystem responses were observed during compound droughts when both SSMI and SAMI signalled extreme droughts. Such conditions were occurring at the study sites on only a handful of days during the last decade, except in 2018, when both SAMI and SSMI were extreme at SE‐Htm for 33 days (Figure S9). Similar long lasting compound droughts had not been observed at the site prior the Year 2018. By comparing Figures 8, S8 and S9 with Figure 7, it is evident that frequent occurrence of extreme droughts had an impact on annual flux estimates.

The frequency of SSMI and SAMI extremes differs because VPD exhibits strong day to day variability due to synoptic scale weather patterns, whereas soil moisture is an integrated measure of the ecosystem water balance and thus varies more slowly than VPD (Figure 3a). This means long (approximately 60 days, depending on location) atmospheric droughts were typically needed for the formation of extreme soil droughts (SSMI < −2) (see Figure S10). One should, however, note that SSMI and SAMI indices are based on empirical CDFs; therefore, a single very extreme summer (e.g., 1959 at SE‐Nor for SSMI) can alter the CDF in such manner that low index values are not reached at other years. This is since the tails of the empirical CDF remain uncertain. We opted to use SSMI and SAMI in this work instead of more traditional drought metrics such as standardized precipitation index (SPI, McKee et al. 1993) or standardized precipitation evapotranspiration index (SPEI, Vicente‐Serrano et al. 2010) since SPI and SPEI require a predefined data aggregation time, and this choice is often arbitrary and possibly varies between locations. In addition, SPEI ignores the effect of snow accumulation and melt, a key regulator of summer soil moisture dynamics at the high latitudes (e.g., Trautmann et al. 2018; Nousu et al. 2024). In contrast, SAMI and SSMI are based on daily VPD and soil moisture known to directly affect the coupled ecosystem C, water and energy cycles. Moreover, our drought indices represent timescale relevant for the flux variability and require no additional temporal aggregation. The use of simulated soil moisture for characterizing droughts has been criticized (Samaniego et al. 2013); however, ERA5‐Land soil moisture product has shown relatively good agreement with observations in past validation studies (e.g., Beck et al. 2021; Muñoz‐Sabater et al. 2021; Xue and Wu 2024) and in our comparisons against in situ derived SSMI at FI‐Hyy (see Supporting Information). The use of simulated instead of measured VWC has also the advantage that it ignores methodological challenges (sensor drift, replacements) associated with long‐term soil moisture monitoring (Rasheed et al. 2022). Nevertheless, ERA5‐Land soil moisture should not be considered as an accurate estimate of local soil moisture at a given forest stand, but rather it provides a landscape scale estimate for the soil moisture.

We calculated SAMI and SSMI from “raw” VPD and VWC time series without detrending or deseasonalizing done; for example, in Pohl, Rakovec, et al. (2023). We opted for this approach since our primary purpose of the indices is to explore the impacts of atmospheric and soil droughts on ecosystem fluxes. For this, detecting temporary flux anomalies as deviations from typical thermal growing season behavior (as done here) is more meaningful than analyzing deviations from time series from which a varying baseline (e.g., seasonal variation) is already removed. Boreal forests GPP, Reco and energy partitioning have strongly different temporal patterns over the thermal growing season (Launiainen 2010; Ueyama et al. 2013; Launiainen, Kieloaho, et al. 2022). This is because boreal forests have strong phenology and tend to acclimate to their local mean environmental conditions (e.g., Choat et al. 2012; Franklin et al. 2020; Harrison et al. 2021) and therefore also drought indices used to analyze flux anomalies must describe how the conditions depart from such local baseline conditions (see Supporting Information).

#### 3.3.2 Regional Occurrence and Trends

**FIGURE 9:**
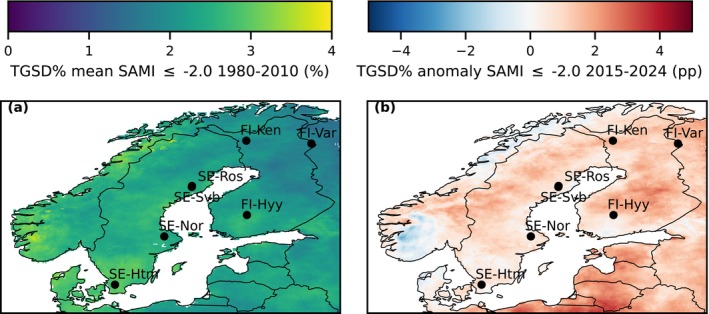
(a) Mean fraction of thermal growing season days (TGSD) in 1980–2010 of extreme atmospheric droughts (SAMI < −2.0) in the Nordic and Baltic countries. (b) Difference in percentage points (pp) between mean the fraction of extreme atmospheric droughts during the last decade (2015–2024) and 1980–2010 reference period. Red color suggests that the occurrence of atmospheric droughts has increased. Map lines delineate study areas and do not necessarily depict accepted national boundaries.

**FIGURE 10:**
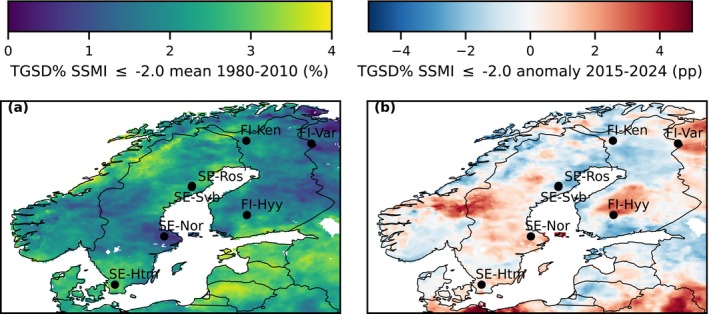
Same as Figure 9 but for mean fraction of growing season days when soil moisture is low (SSMI < −2.0). Red color suggests that the fraction of extreme soil drought days has increased. Map lines delineate study areas and do not necessarily depict accepted national boundaries.

Using gridded ERA5‐Land data, we assessed the spatial patterns of extreme atmospheric (SAMI < −2.0; Figure 9) and soil (SSMI < −2.0; Figure 10) droughts in the Nordic‐Baltic region and explored how their occurrence has changed during the last decade. During 1980–2010 $\left(2.3\pm 0.3\right)\%$ (mean ± std) of the thermal growing season days were characterized dry (SAMI < −2.0), with more frequent occurrence in Denmark, Southern Sweden and coastal Norway and less frequent in the eastern and northeastern Finland (Figure 9a). During the last decade, the fraction of high VPD thermal growing season days has clearly increased in most parts of the area (Figure 9b); also, at the location of the EC sites used in this study (Figure S8). On average this increase has been $\left(1.1\pm 0.8\right)\;\mathrm{pp}$ and strongest in central Finland and the Scandes. Considering that due to climate warming, the average thermal growing season length has increased from $\left(158\pm 43\right)\;\text{days}$ to $\left(168\pm 42\right)\;\text{days}$, this increase means that on average the number of days during thermal growing season when SAMI ≤ −2.0 has increased from 4 days to 6 days per year. Locally, this increase has been at maximum 10 days.

**FIGURE 11:**
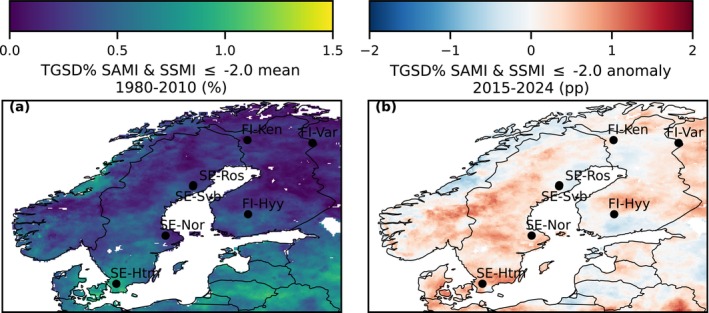
Same as Figure 9 but for mean fraction of growing season days when both atmospheric vapor pressure deficit and soil moisture is low (SAMI < −2.0 and SSMI < −2.0). Note different color scale from Figures 9 and 10. Map lines delineate study areas and do not necessarily depict accepted national boundaries.

The occurrence of extreme soil droughts (SSMI < −2.0) shows much stronger spatial variability than atmospheric droughts (Figure 10a). During 1980–2010, the Baltic countries, Scania, northern Scandes and southern coast of Finland have experienced the most frequent extreme soil droughts (> 3 pp). Compared to the long‐term average, the last decade has been particularly dry in part of central Finland, central Norway and central and southern parts of Sweden (Figure 10b). Finally, compound droughts, that is, periods when both SAMI < −2.0 and SSMI < −2.0, are rarer than atmospheric or soil drought alone (Figure 11a). The compound drought anomaly (Figure 11b) is smaller compared to the either of the droughts alone and show no clear pattern in Finland or the Baltics. In southern Sweden, southern Norway and Denmark the compound drought shows small increase (ca. 1–2 pp) during the recent years. This converts to a few growing season days on average per year, or alternatively to a longer dry spell once or twice per decade, similarly as in the case of in the increase of SAMI ≤ −2.0 days.

Overall, the results suggest extreme atmospheric droughts have become more frequent over the whole region during the last decade, while changes in extreme soil and compound droughts are more local and no trend covering the whole region can be detected. Note that these results differ with the recent analyses on European soil and atmospheric droughts by Shekhar, Buchmann, et al. (2024). Their results suggest that soil moisture has an increasing trend in Northern Europe and that, for example, in Finland atmospheric droughts have become less common. The differences arise particularly from different reference and present periods. Shekhar, Buchmann, et al. (2024) analyze the SWC and VPD extreme shifts between reference (1950–1990) and target (1990–2021) periods that are longer than in this study (reference: 1980–2010, target: 2015–2024).

While extreme soil drought conditions during which carbon fluxes were decreased have been rare (Figures 8 and 10), their occurrence may increase in the future. If long‐lasting atmospheric droughts increase in the future, for example, due to increased occurrence of persistent weather phenomena such as the anomalously high‐pressure system during the Year 2022 over France (van der Woude et al. 2023), also extreme soil droughts may also become more common. In Supporting Information it is shown that approximately 60 days of continuous atmospheric drought is needed for creating significant soil droughts at the study sites.

### 3.4 Can Droughts Have Contributed to the Recent Growth Decline of Nordic Forests?

Our results provide important insights on northern boreal forest C and water flux response to atmospheric and soil droughts based on seven EC sites (Table 1). We observed a consistent decrease of daily GPP and NEP during periods of high VPD, as well as during the rare cases of soil drought (Figure 6; Figures S1–S6). Analysis of the regional changes in atmospheric (SAMI) and soil drought (SSMI) indices further indicated that high VPD conditions have become more common across the whole Nordic‐Baltic region during the last decade compared to the 1980–2010 reference period (Figure 9). Extreme soil droughts and changes in their frequency, however, have been more local and a large‐scale trend is lacking (Figure 10). Our results further showed drought‐induced decrease of annual GPP of approximately 10% in extremely dry years (Figure 7), however, such strong reductions were rare.

Beyond remaining case studies over a small number of sites, EC data has inherent limitations in its capacity to assess tree growth variability. Firstly, the EC towers can capture the inter‐annual NEP variability, from which annual GPP anomalies can be derived. However, moving from drought‐induced changes in GPP to variability in tree net primary production (NPP), the amount of C available for growth, requires understanding how, for example carbon use efficiency (CUE = 1−*R*_*a*_/GPP, where *R*_*a*_ is autotrophic respiration) and partitioning of GPP between trees and understory vary during drought (e.g., Liu et al. 2019; Martínez‐García et al. 2024). Secondly, stem growth and carbon uptake can show different responses to environmental drivers such as droughts (Cabon et al. 2022), suggesting that the controls of ecosystem‐atmosphere carbon fluxes observed here on VPD, and soil moisture do not necessarily have direct correspondence with the controls of tree growth. Thirdly, it is hypothesized that trees allocate more resources to root growth in water‐limited conditions (Brunner et al. 2015). The large‐scale growth decline observed across the Nordics is primarily derived from the observed decline of basal area growth (Henttonen et al. 2024; Laudon et al. 2024), which does not enable assessing potential changes in carbon allocation. On the other hand, the EC technique observes the carbon fluxes between the whole ecosystem and the atmosphere; hence NEP and GPP are insensitive to changes in carbon allocation if they do not alter ecosystem scale fluxes; for example, via increased soil respiration. Therefore, the increased carbon allocation to roots in response to droughts could be interpreted as decreased stem growth, whereas such changes in allocation patterns are not reflected in EC GPP observations.

Finally, extreme environmental conditions are, by definition, rare, and long time series are required to gain good statistics on their effect on ecosystem function. Luckily, decades‐long EC flux time series are beginning to become available (e.g., Pilegaard and Ibrom 2020; Launiainen, Katul, et al. 2022; Grünwald et al. 2025; Krebs et al. 2025). Nevertheless, evaluating daily patterns of VPD and soil water availability against their long‐term local climatic patterns using the novel approach based on SAMI and SSMI indices developed in this study will enable assessing whether EC observations done over a short period; for example, a couple of years, represent local extremes or typical environmental conditions. This would be impossible to evaluate from the short time series alone.

While ecosystem GPP anomalies are not directly transferable to growth variability, a significant positive correlation between them exists (Cabon et al. 2022). Our results enlighten the role of atmospheric and soil droughts behind the enigmatic forest growth decline recently observed across the Nordic countries (Henttonen et al. 2024; Korhonen et al. 2024; Mensah et al. 2023; Merlin et al. 2024). In Finland, growth of Scots pine has declined mostly in the northern part of the country, consistently both on mineral soil and drained peatlands (Henttonen et al. 2024; Korhonen et al. 2024; Helama 2024). At the same time, growth of Norway spruce has shown no decline (Henttonen et al. 2024). If the growth decline would be purely drought‐related, it would imply that large‐scale hydroclimate has become more unfavourable for Scots pine particularly in the Northern Finland, while Norway spruce or deciduous species are not affected. Our results show that high VPD events are frequent (Figure S8) and there has been uniform increase of extreme atmospheric droughts in the Nordic‐Baltic region during the last decade (Figure 9). Noting the stronger VPD sensitivity of stomatal conductance (e.g., Oren et al. 1999) and non‐stomatal limitations of photosynthesis (Salmon et al. 2020) in Scots pine compared with Norway spruce, elevated VPD could contribute to the observed species‐specific growth responses (Henttonen et al. 2024). In large parts of Canada, decline of boreal forest growth has been attributed to warming‐induced increase of VPD (Mirabel et al. 2023). At the same time, strong spatial variability of soil water limitations is proposed as the reason for the diverging forest productivity trends between western and eastern Canada (Ma et al. 2012). Similar contrasts emerge also between Finland and Sweden (Henttonen et al. 2024; Mensah et al. 2023). In Sweden, growth reduction of Norway spruce has been stronger than that of Scots pine (Mensah et al. 2023), especially in Southern Sweden. This could be due to more frequent soil water limitations in the region (Figure 10; Goude et al. 2022) and the known sensitivity of spruce to soil drought (Lévesque et al. 2013; Aldea et al. 2024). In Norway, Merlin et al. (2024) report that the correlation between annual diameter growth and temperature and soil water availability vary strongly across regions. Despite last decades of warming, the climate‐growth relationships have remained stable, and soil droughts are so far considered to be insignificant for forest growth in Norway (Merlin et al. 2024). Our results show that soil droughts have been rare at the seven EC sites studied, and there has not been a uniform trend in the occurrence of extreme soil droughts in the last decade (Figure 10). It is thus unlikely that widespread soil moisture limitations could underlie the large‐scale growth decline in the Nordic countries. Especially, soil droughts are unlikely to explain the observed growth decline in Northern Finland (Mäkinen et al. 2022). Other factors, such as changes in forest age structure, may also play a role in explaining the growth decline (Henttonen et al. 2024; Mäkinen et al. 2022). So far, the role of environmental change on the growth decline observed in NFI data has been addressed only at regional scale (e.g., Mäkinen et al. 2022; Helama 2024). While this may be viable for addressing the effects of temperature and VPD changes driven by large‐scale hydroclimatic change, strong heterogeneity of boreal forest landscape due to topography, soils and fertility amplifies spatiotemporal variability of soil moisture (Larson et al. 2022; Launiainen, Kieloaho, et al. 2022; Nousu et al. 2024), making soil drought inherently a local feature. As soil moisture limitation of photosynthesis and water use is a highly non‐linear process, it is necessary that growth variability in future studies is analysed at a spatial scale (e.g., NFI plot or small region) where soil moisture (or its proxies such as P–ET) variability is not blurred by spatial averaging.

## 4 Conclusions

The C sink of boreal forests in the Nordic countries has declined over the last decade, and increased water limitations on forest growth have been proposed as a potential, yet speculative reason for the phenomenon (Laudon et al. 2024). We used long‐term eddy‐covariance time series from seven (hemi)boreal forests in Sweden and Finland to assess how atmospheric droughts (elevated VPD) and soil droughts (limited soil water availability) have affected ecosystem productivity and water use. To provide insights on this, we developed new statistical metrics to relate daily VPD (SAMI) and soil moisture (SSMI) conditions to their respective average seasonal cycle during the thermal growing season. We further showed that anomalies in NEP, GPP, Reco, and evaporative fraction can be linked to extreme values of SAMI and SSMI. We found that soil moisture has limited boreal forest ecosystem functions and C and water fluxes at the study sites only at very extreme and hence infrequent cases. In contrast, increasing VPD decreased GPP and apparent photosynthetic capacity over a wide range of conditions, implying frequent VPD limitations. Strongest ecosystem flux anomalies were observed during compound droughts when both air and soil moisture were low.

On annual scale, significant impacts of atmospheric and soil droughts were observed only at a few isolated years and the drop in annual GPP was ca. 10% at maximum, indicating that persistent drought‐induced decline in GPP or NEP has not taken place at the study sites. For instance, during the 20‐year time series from a mixed coniferous forest stand (FI‐Hyy) in Southern Finland only 3 years (1999, 2006 and 2018) stand out as drought years. Nevertheless, based on ERA5‐Land data, a widespread increase in the occurrence of strong atmospheric droughts were found across the Nordic and Baltic countries, suggesting VPD limitations on ecosystem carbon uptake have likely increased when compared to prior decades. Atmospheric droughts have also been projected to become more frequent with climate warming (Novick et al. 2016) and hence VPD limitations on forest growth may become more prevalent in the future.

Our study provides a significant methodological improvement for investigating how ecosystems respond to extreme droughts, and for estimating the contribution of drought‐related anomalies to annual NEP, GPP and Reco. Firstly, the framework utilising both atmospheric (SAMI) and soil (SSMI) drought indices, developed in this study, identify extreme drought conditions directly from statistics of the controlling environmental variables, that is, VPD and soil moisture. This is clear improvement over traditional indirect approaches, such as the use of SPEI. Secondly, our approach to identify drought‐induced daily anomalies from flux time series can be used to delineate water stress responses from all EC sites globally, across different ecosystems and climate zones. Use of well‐documented response curves (e.g., photosynthetic light response) rather than correlative models (e.g., machine learning models) to delineate the anomalies has the benefit of being less susceptible to the inadvertent removal of the studied responses from the anomalies. Third, the novel conditional distribution sampling technique to quantify the effect of temporary drought anomalies on annual C fluxes significantly improves our capacity to isolate the effects of drought responses from other inter‐annual variability in flux data.

Our finding that atmospheric droughts have restricted forest carbon uptake at the seven studied (hemi)boreal forests aligns with Mirabel et al. (2023) who attributed the decrease of boreal forest growth in Canada to warming‐induced increase of VPD. To reveal how atmospheric (SAMI), soil (SSMI) or their co‐occurrence has affected tree growth during the past decades across the Nordic‐Baltic region, systematic analysis of tree ring width chronologies and plot‐level growth data from National Forest Inventories against drought indices SSMI and SAMI is necessary.

## Author Contributions

**Samuli Launiainen:** conceptualization, writing – original draft, writing – review and editing, funding acquisition. **Olli‐Pekka Tikkasalo:** conceptualization, visualization, methodology, formal analysis, writing – review and editing, data curation, software, writing – original draft. **Jari‐Pekka Nousu:** formal analysis, writing – review and editing, visualization. **Olli Peltola:** conceptualization, methodology, visualization, writing – original draft, data curation, funding acquisition, software, formal analysis, writing – review and editing. **Janne Rinne:** conceptualization, writing – review and editing, funding acquisition.

## Funding

This research has been supported by the EU Horizon 2020 Framework Programme, EU H2020 Excellent Science (GreenFeedBack, grant no. 101056921) by European Commission Just Transition Fund, through Council of Oulu Region (2021/900302/09), and the Research Council of Finland (grant nos. 356138, 354298).

## Disclosure

The views and opinions expressed are those of the author(s) only and do not necessarily reflect those of the European Union. Neither the European Union nor the granting authority can be held responsible for them.

## Conflicts of Interest

The authors declare no conflicts of interest.

## Supporting information


**Data S1:** gcb70962‐sup‐0001‐DataS1.pdf.

## Data Availability

Python code to reproduce the figures can be found in Zenodo (https://doi.org/10.5281/zenodo.20494450). NetCDF files containing the drought indices SAMI and SSMI between 1950 and 2025 for Northern Europe are also in Zenodo (https://doi.org/10.5281/zenodo.19885257). Persistent identifiers to the used eddy covariance observations: FI‐Hyy (http://urn.fi/urn:nbn:fi:att:af0b5d17‐6630‐43a6‐acf8‐223064a8bd82), FI‐Ken (https://hdl.handle.net/11676/l_qfCXRuMUS2nFMSvmWmfucQ), FI‐Var (https://doi.org/10.23729/cbd23aef‐04b8‐44a7‐b05d‐4e8e64ad6d69), SE‐Nor (https://hdl.handle.net/11676/f1tLJAI708YA5w37gX6UNQbY), SE‐Htm (https://hdl.handle.net/11676/YJkrKWJx317TTXKmMk4XZRif), SE‐Svb (https://hdl.handle.net/11676/ujM7vxk12vKCoquLSToLG18z) and SE‐Ros (https://hdl.handle.net/11676/N5q7xRpGuvY95jyTgphht6ZA).
