# Supplementary material for: Impacts of Atmospheric and Soil Droughts on Carbon and Water Fluxes in Northern European Coniferous Forests: Evidence From Eddy‐Covariance Data
Source: Glob Chang Biol. 2026 Aug 5;32(8):e70962. doi: 10.1111/gcb.70962 (PMC13439358; doi:10.1111/gcb.70962)
Supplement: Supplementary file 1 [file GCB-32-e70962-s001.pdf]

Impacts of atmospheric and soil droughts on carbon and water fluxes in northern European coniferous forests: evidence from eddy-covariance data

Olli Peltola[1], Olli-Pekka Tikkasalo[1], Jari-Pekka Nousu[1], Janne Rinne[1], Samuli Launiainen[1]

[1]Natural Resources Institute Finland, Latokartanonkaari 9, 00790 Helsinki, Finland

Correspondence to: Olli Peltola (olli.peltola@luke.fi)

Supplementary material

Fig. 6 for other sites than FI-Hyy

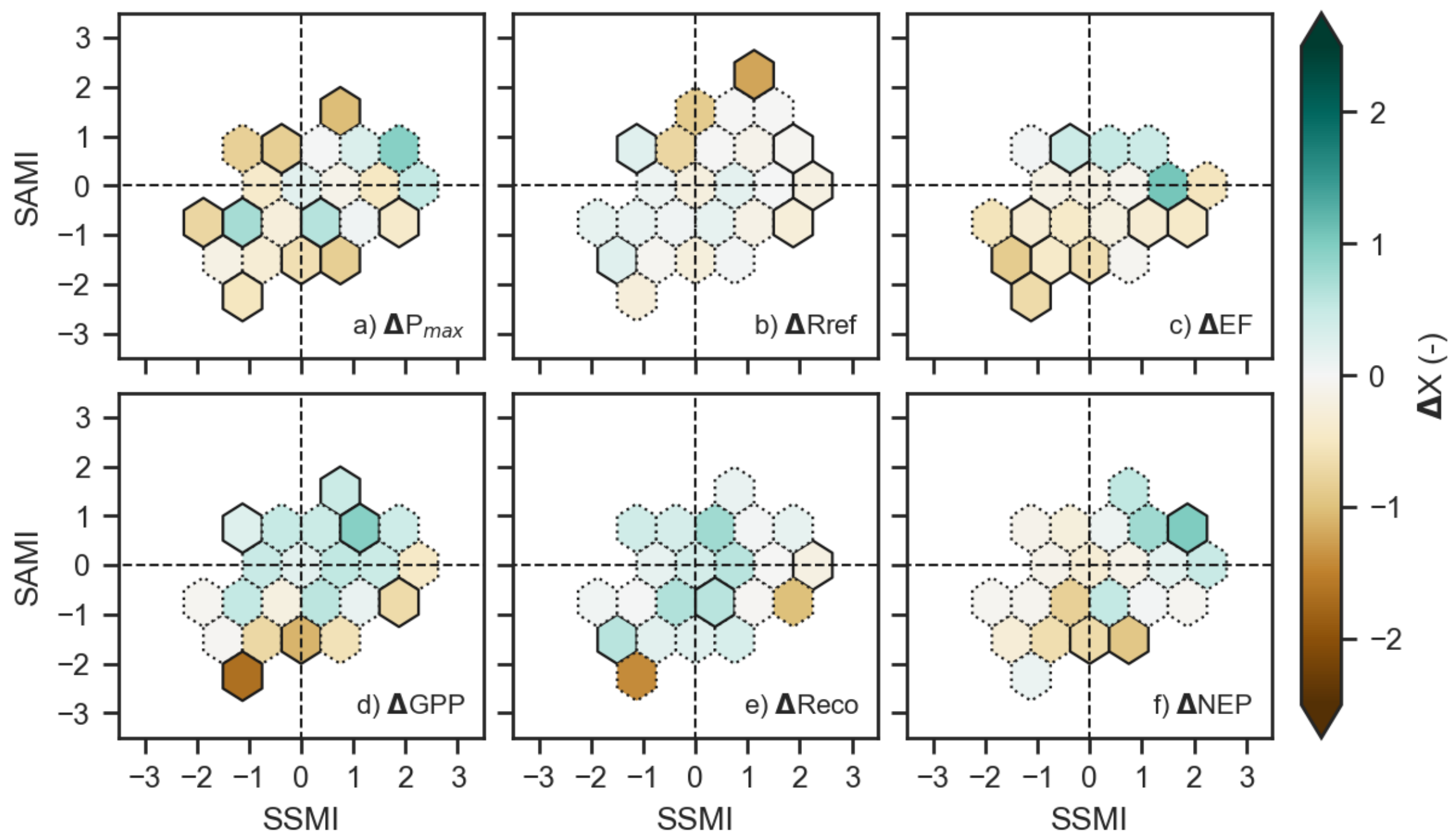

Fig S1. Similar figure as Fig. 6, but for SE-Svb.

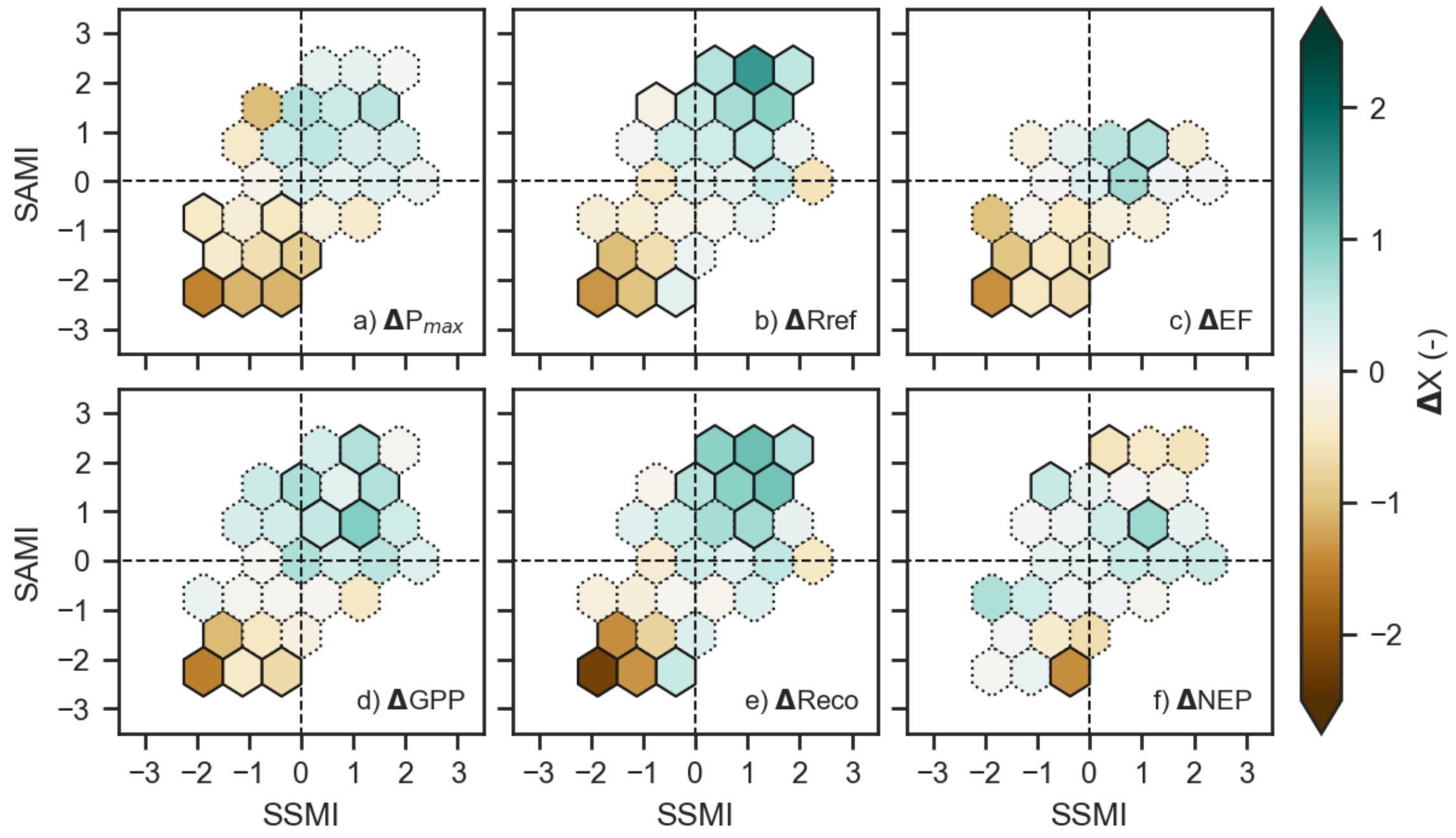

Fig S2. Similar figure as Fig. 6, but for SE-Ros.

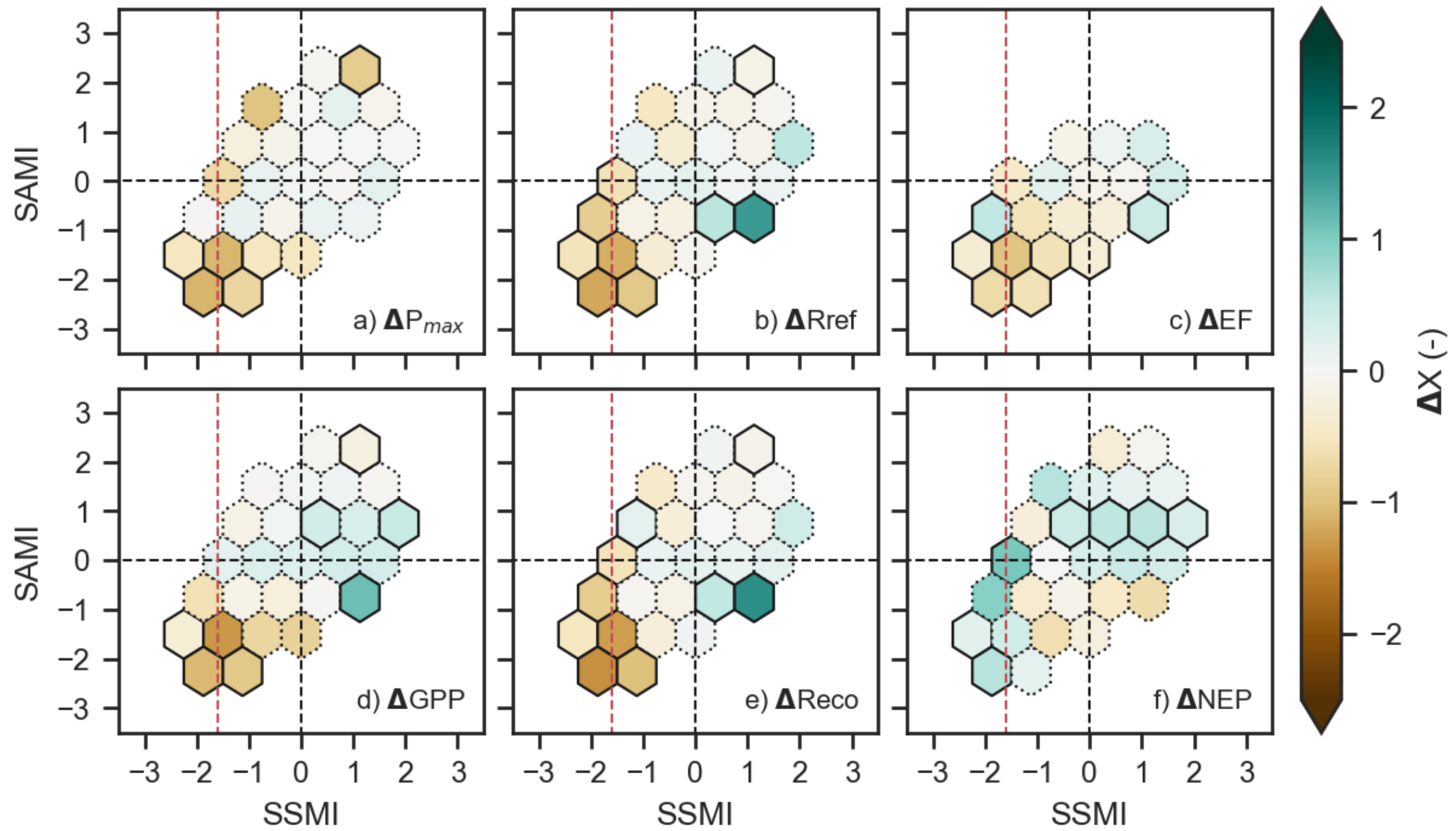

Fig S3. Similar figure as Fig. 6, but for SE-Nor.

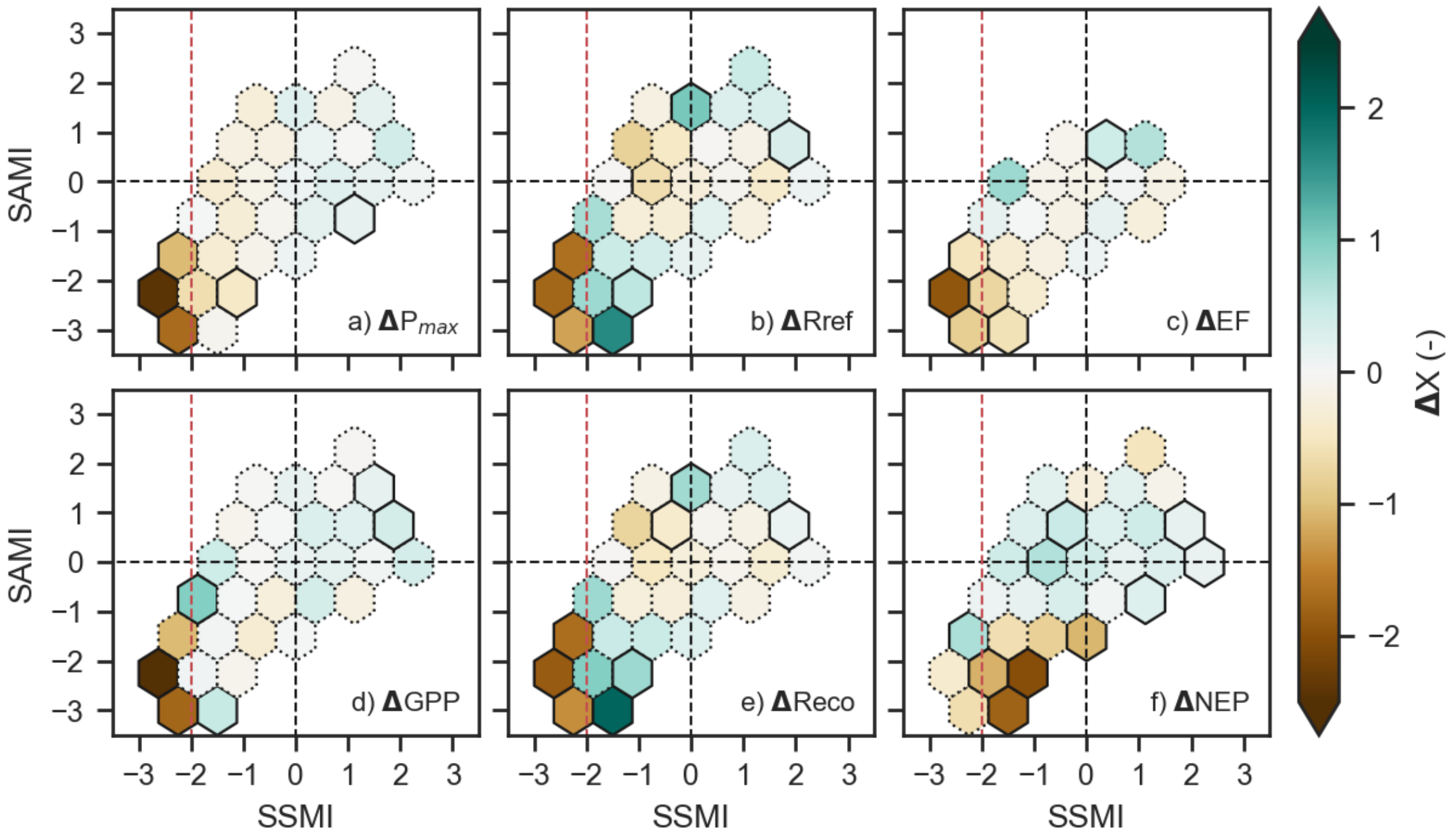

Fig S4. Similar figure as Fig. 6, but for SE-Htm.

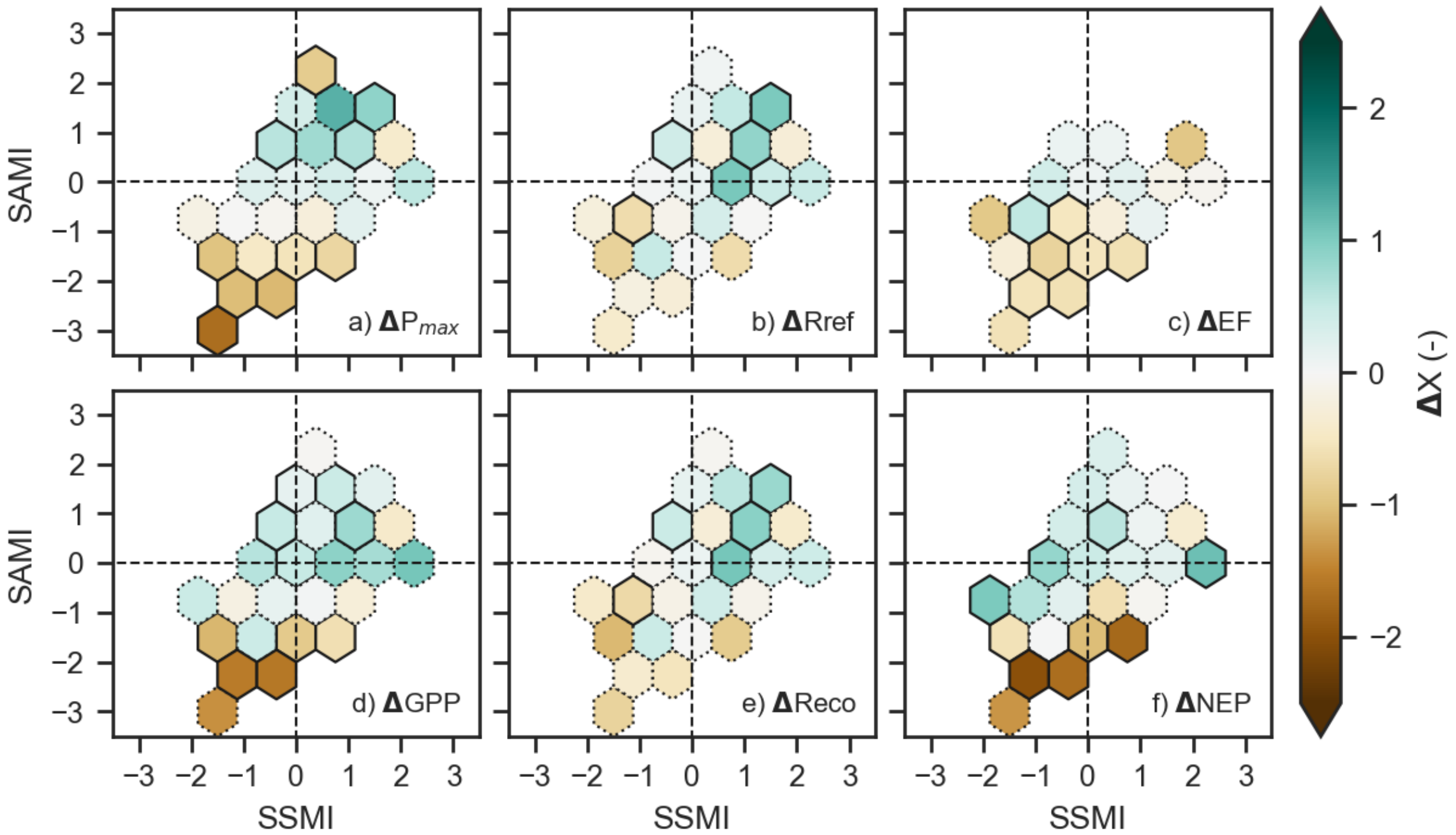

Fig S5. Similar figure as Fig. 6, but for FI-Ken.

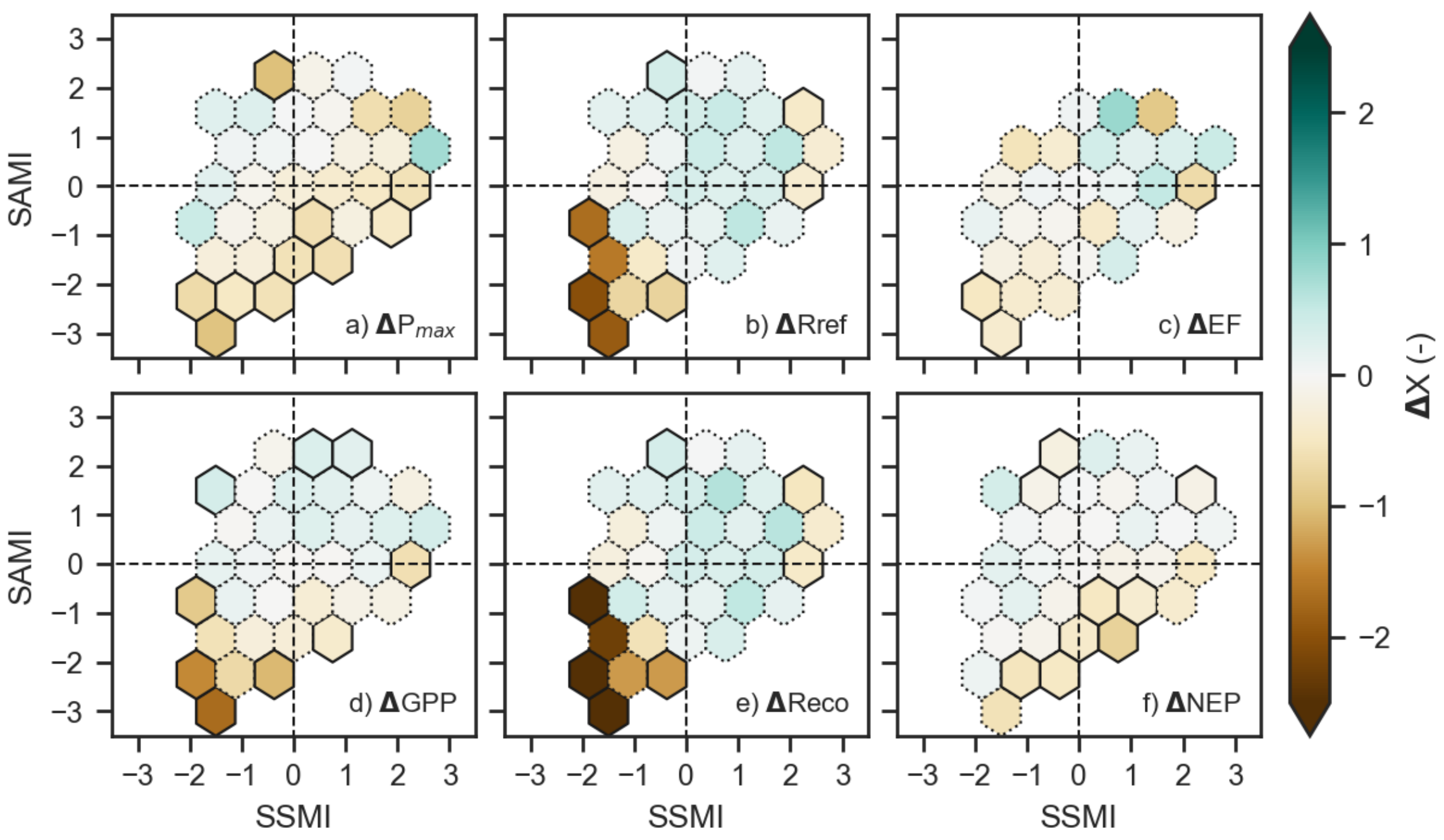

Fig S6. Similar figure as Fig. 6, but for FI-Var.

Effect of deseasonalising on drought indices

Derivation of the drought indices used in this study was guided by the question: what are extreme conditions for the studied ecosystems? If the forest stands have acclimated to the typical local water availability conditions, as suggested e.g. by Choat et al. (2012), then the used indices should be able to distinguish how much the conditions differ from these typical local conditions in order to be viable metrics for assessing the ecosystem drought responses. This reasoning motivated us to derive the drought indices SSMI and SAMI without deseasonalising or detrending, i.e. use the VWC and VPD as they were for the calculation of the index values. With this approach SSMI and SAMI describe how much the local conditions differed from median local conditions observed during the growing seasons between the years 1950 and 2020. Hence, with this definition the median growing season conditions (VPD for SAMI and VWC for SSMI) serve as a baseline against which the departures are evaluated with SAMI and SSMI, i.e. indices equal zero at median growing season conditions. However, the trees and other parts of the ecosystem can actively adapt their anatomy to changing conditions and utilising long-term median conditions as a baseline misses this effect. The trees can e.g. alter the root anatomy and biomass to enhance water uptake from the soil or reduce leaf area to minimise water loss (e.g. Brunner et al., 2015; Novick et al., 2024). Yet, the time scale at which these anatomical alterations in response to drought take place is unclear and likely depend on species and other limitations to growth besides water availability (e.g. nutrient availability, light, soil type). Hence, for simplicity we opted to utilise the median growing season conditions as a baseline against which the indices were estimated. However, ideally the baseline should be calculated at such time scale that corresponds to the time scale at which plants acclimate to the changing conditions via these anatomical alterations. If the time scale would be known, then the VWC and VPD could be filtered e.g. with running median filter before estimating the indices.

Viewed from this perspective, deseasonalising the VPD and VWC time series before calculating the indices SSMI and SAMI or doing the index calculation separately for each day of year (e.g. Pohl et al., 2023), is not necessarily advisable. This is because the ecosystems are not likely acclimated to mean seasonal variability, but rather to longer term local conditions. For instance, it is difficult to conceive a root-to-shoot ratio for a tree that is optimal separately for each day of year, rather than longer term average conditions. However, plant phenology has a seasonal pattern and hence especially when analysing drought responses of forests dominated by deciduous trees it might be advisable to use deseasonalising before calculating the indices. For instance, the response of an ecosystem to extreme VPD might be different when leaves are out than during leafless period. However, this is partly considered by focusing on thermal growing season when calculating the indices.

Nevertheless, we evaluated how the SAMI and SSMI values would change if VPD and VWC time series would be deseasonalised and detrended before calculating the indices. We utilised similar deseasonalising and detrending strategy as for FI-Hyy and FI-Var flux data (see Sect. 2.2 in the main text) and compared the indices calculated from these filtered time series ($SSMI_d$ and $SAMI_d$) against the indices used otherwise in this study (SSMI and SAMI). Figs. S6 and S7 show the comparisons for two example sites FI-Hyy and SE-Svb. There is scatter between the indices calculated with or without deseasonalising, especially in the wet conditions (index values above 0). The agreement has a seasonal pattern, for instance $SSMI_d$ suggest moderate drought (index values below 0) occasionally during the first part of summer, whereas SSMI is near zero or above during these periods. Conversely, $SAMI_d$ is occasionally below 0 during late growing season when SAMI is positive. However, the indices with or without deseasonalising largely agree when they are strongly negative, i.e. dry conditions. This suggests that drought analyses using SSMI and SAMI are not very sensitive to the fact that whether SWC and VPD are deseasonalised before calculating the index values.

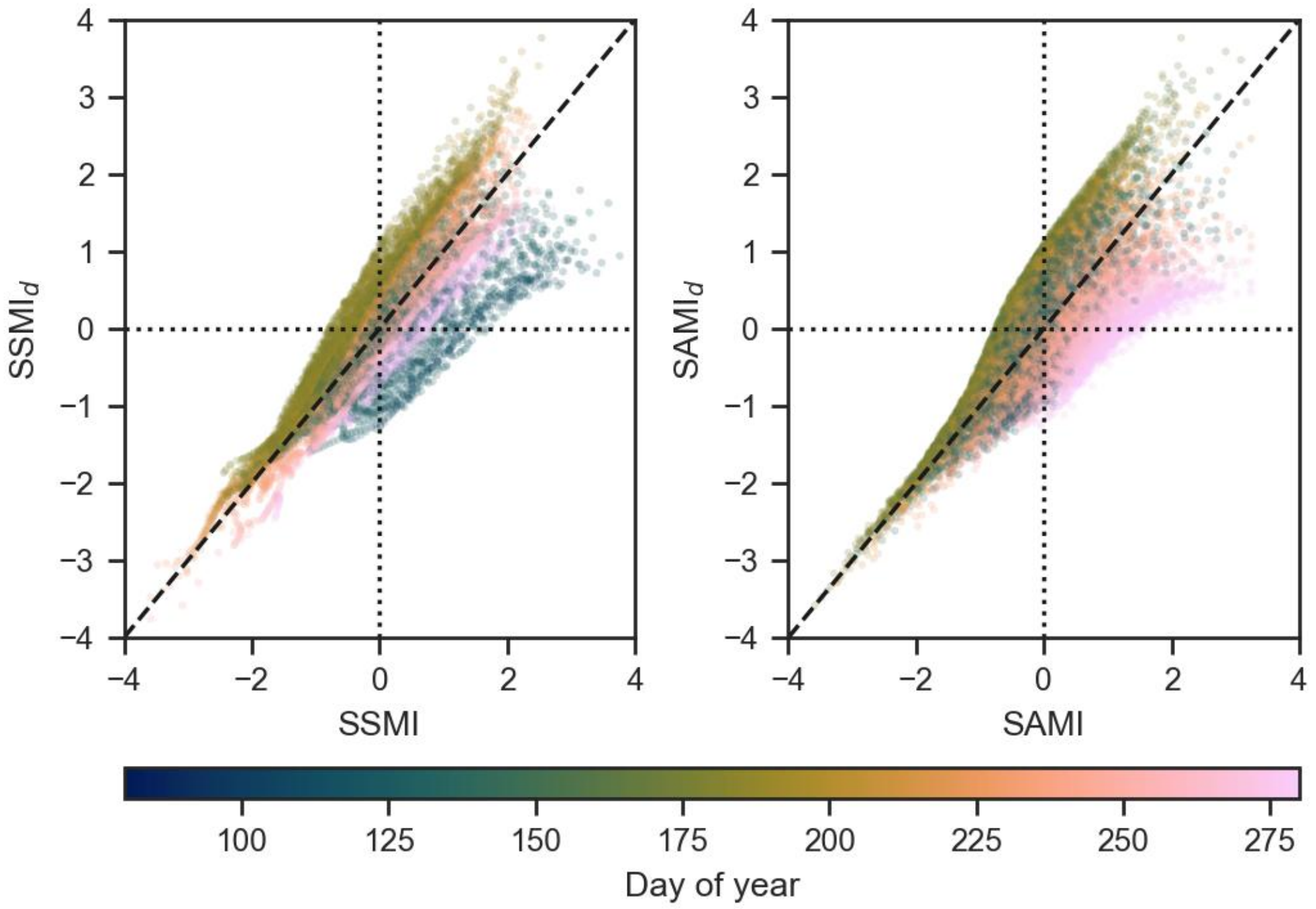

Fig S6. Comparison between SSMI and SAMI calculated with (denoted with subscript d) or without deseasonalising VWC and VPD time series, respectively, before calculating the index values. This figure shows data from FI-Hyy between the years 1950 and 2020. Black dashed line shows 1:1 line.

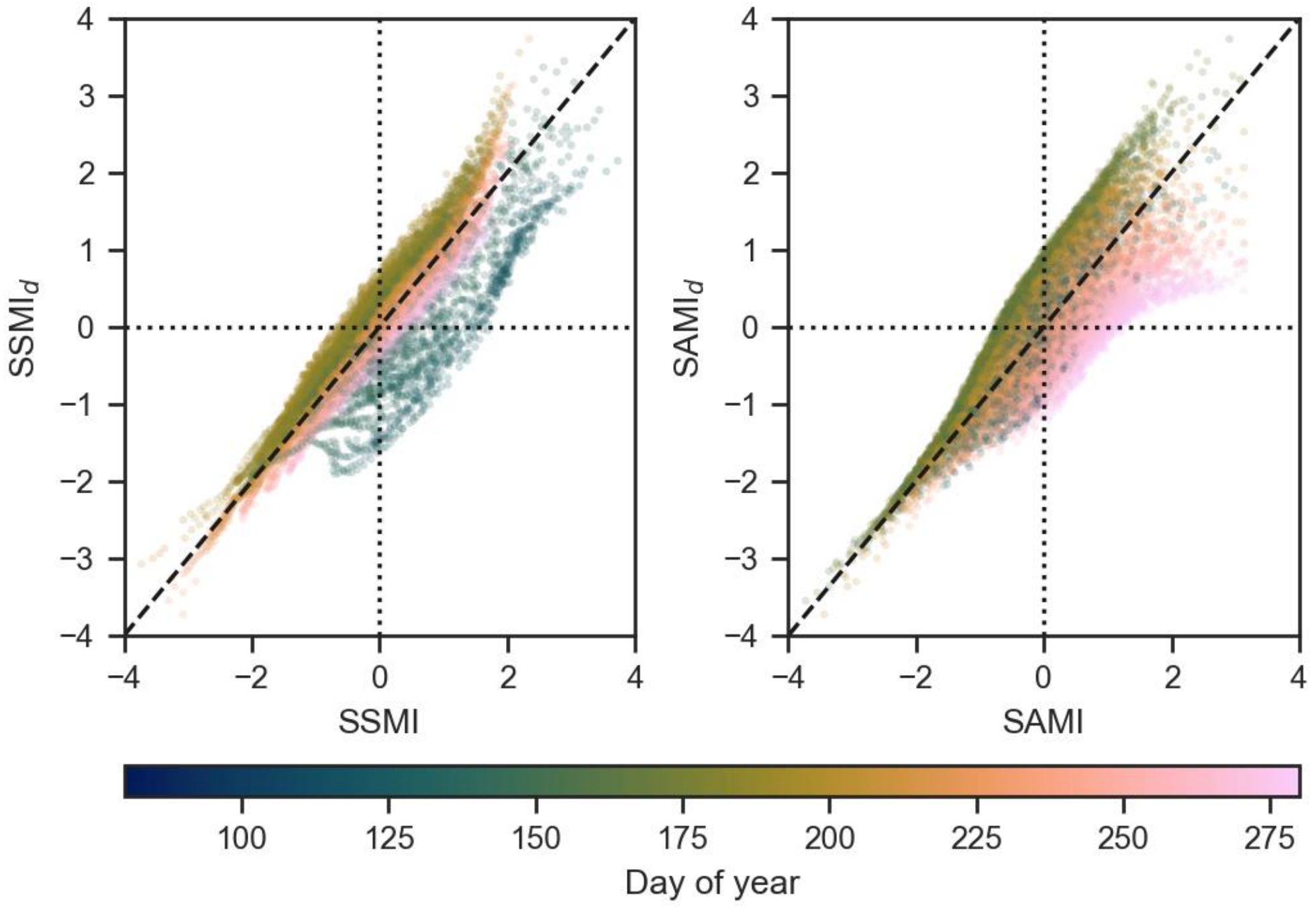

Fig S7. Similar figure as Fig. S6, but for SE-Svb.

Occurrence of atmospheric and compound droughts at the study sites

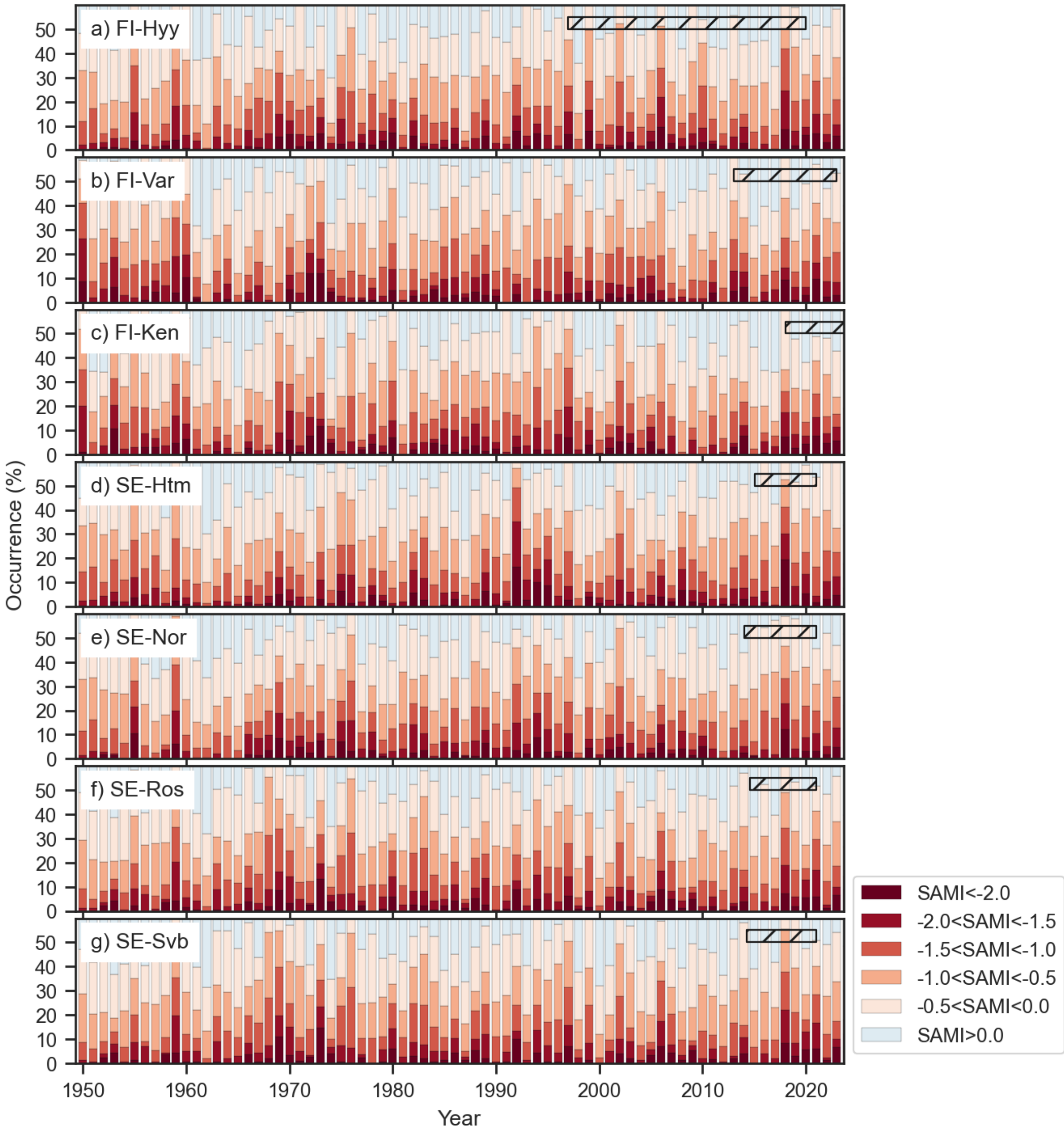

Fig S8. Same as Fig. 8 in the main text, but for air moisture index SAMI.

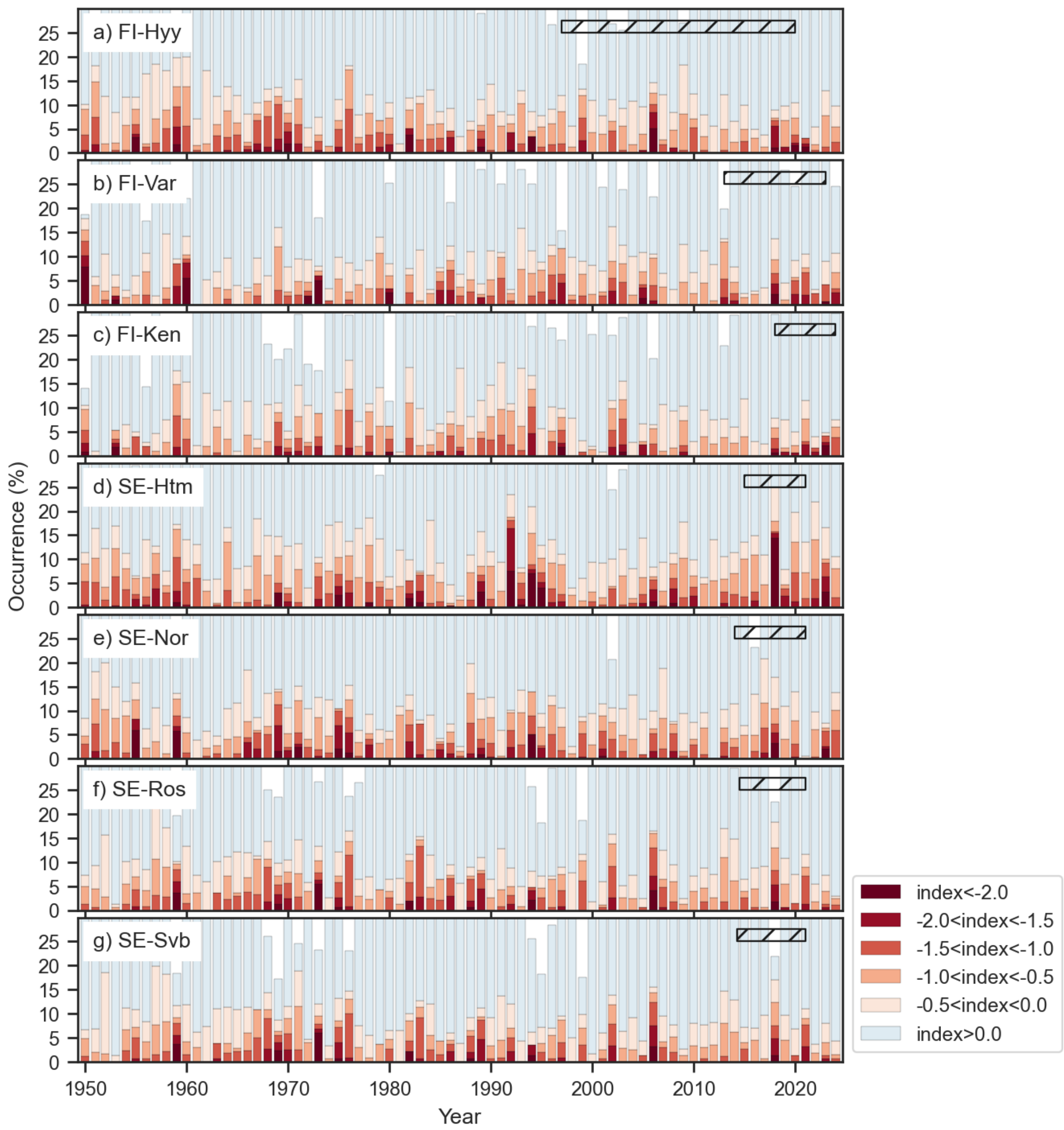

Fig S9. Same as Fig. 8 in the main text, but for both SAMI and SSMI. Hence, here the dark red columns show the yearly occurrence of extreme compound droughts (SSMI<-2 & SAMI<-2). Note the difference in y-axis scale when compared to Figs. S8 and 8.

Distribution of SAMI before soil droughts

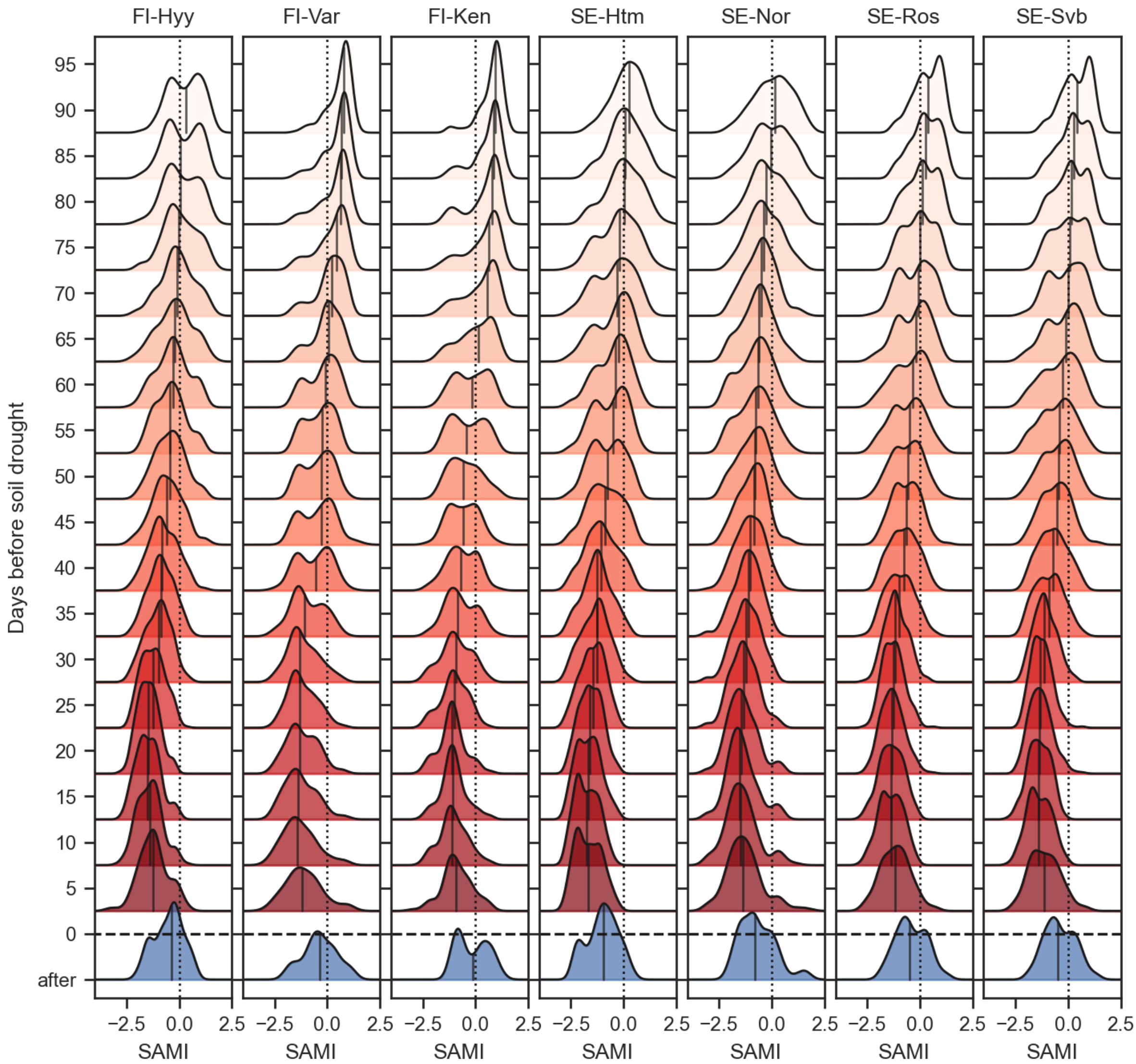

Fig. S10. Distribution of SAMI values during the periods before extreme soil drought (SSMI<-2) was occurring at the EC sites. Distributions of SAMI values during 15 day periods after soil drought are shown in the bottom with blue. Distributions were estimated with kernel density estimation and the vertical black lines within the distributions show the median values. Vertical dotted line shows SAMI=0, i.e. conditions when VPD was equal to the median growing season value at the site during the years 1950-2020.

Comparison of ERA5-Land and in-situ derived SSMI at FI-Hyy

In-situ measured soil moisture data at –5cm, -10cm, and –30cm depths at the FI-Hyy site were obtained from Aalto et al. (2022). for a period of 2018 to 2024. SSMI for each *in-situ* measurement depth were calculated using the same procedure as for the ERA5-Land data. Additionally, to ensure comparability with the *in-situ* measurements, SSMI were recalculated from the ERA5-Land data for the same measurement period. Figure S10 shows time series of SSMI from both *in-situ* and ERA5-Land. Overall, ERA5-Land and *in-situ* SSMI exhibit similar seasonal patterns. However, compared to the *in-situ* measurements, ERA5-Land tends to overestimate temporal variability, showing more pronounced drops during droughts (see e.g., 2018, 2020 and 2021 in Fig. S10) while underestimating their duration (see e.g., 2018, 2019 and 2022 in Fig. S10)

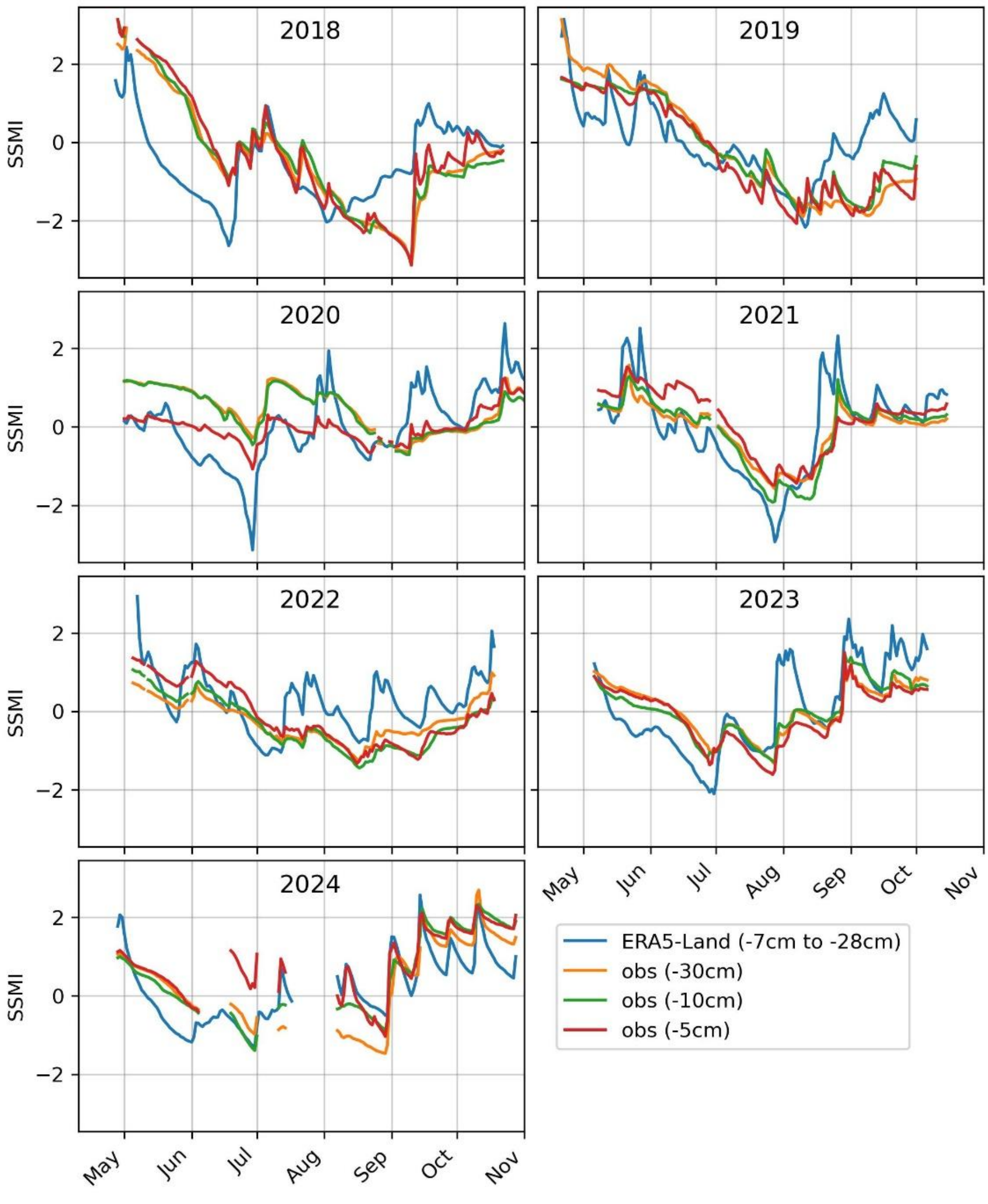

Fig. S11. Time series comparison of in-situ measured SSMI at –5 cm, –10 cm, and –30 cm depths (obs) with ERA5-Land SSMI at FI-Hyy during the growing seasons from 2018 to 2024.

Possible lagged effect of past drought period length on the estimated SSMI-SAMI dependence

The approach estimating drought responses by binning ΔX values in SSMI-SAMI bins assumes that the ecosystem responses to the current SSMI-SAMI state is instantaneous and the past states do not strongly alter the response. We utilized the following procedure to assess the accuracy of this assumption: first, we identified all the periods during which SSMI was below a particular value (see Fig. S12 below for an example) and then evaluated whether ΔX is more negative at the end (termination) than at the beginning (onset) of the period. This is what one would expect if the length of the past drought period would be important, i.e. the past state of the ecosystem (soil drought) would largely alter the current ecosystem response to a particular soil moisture deficit. On the other hand, if the past state is not important, then the current state of the ecosystem dictates the ecosystem responses, which suggests that our approach utilizing SSMI and SAMI is not biased by the fact that we do not consider the past states in our approach.

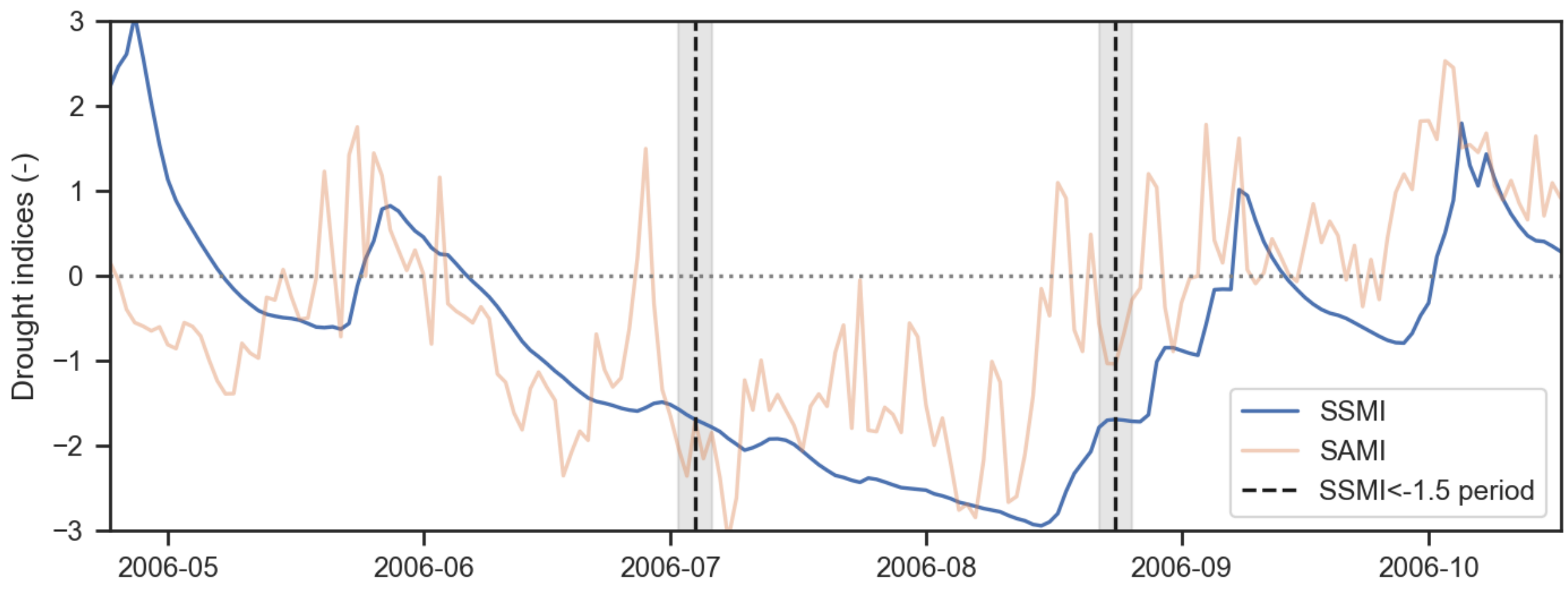

Figure S12. Example of a period during which SSMI was continuously below a specific value (-1.5 in this example). The period onset and termination are shown with vertical dashed lines. The shaded areas show 5-day intervals over which the ΔX was averaged over for calculating its value separately for the onset and termination of that drought period. Data from FI-Hyy were used in this example.

Figure S13 shows results from this analysis for FI-Hyy GPP signal. Contradictory to the expectation, ΔGPP was typically closer to zero at the termination of the drought period than at the beginning of the period (see Figs. S13a, S13b and S13c) suggesting that the soil drought impact on the ecosystem GPP was less severe at the end than at the beginning of the period. At first this seems counterintuitive; however, the difference can be explained with SAMI and the time delay between SSMI (soil moisture) and SAMI (VPD). During the onset of soil droughts SAMI values were negative and the corresponding high VPD was partially driving the gradual decrease of SSMI and soil moisture (see Fig. S12). In contrast, during the termination of the drought period SAMI values varied typically around zero. This is because rainfall and moist air (SAMI close to zero) terminated the soil drought periods. Hence, during the onset of dry soil periods both SAMI and SSMI were negative (compound drought), whereas during the termination, only SSMI was usually negative (single-component drought).

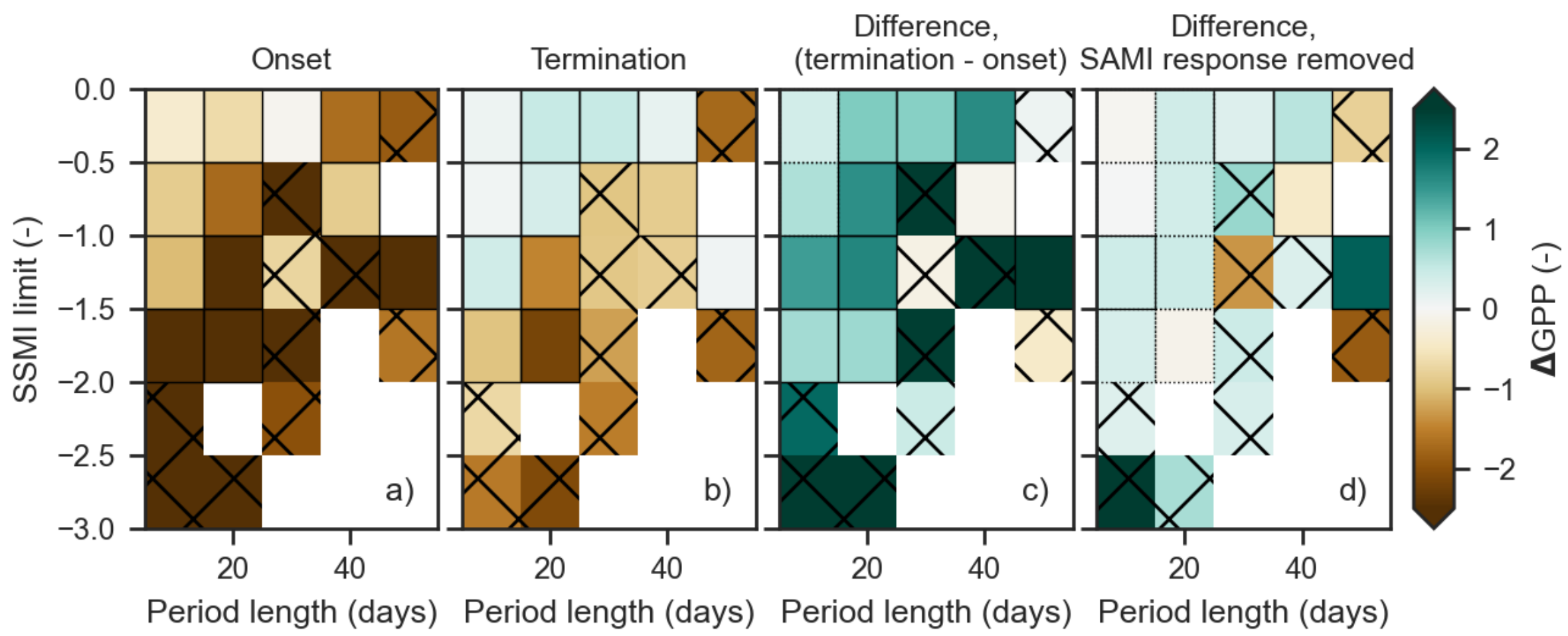

Figure S13. Bin medians of ΔGPP at the onset (a) and termination (b) of periods of different length during which SSMI was below a specific value (SSMI limit). Subplot c shows the difference between these two and in subplot d the ΔGPP response to different SAMI values at the onset and termination are additionally accounted for (see text for details). Hatched areas show bins where less than 5 individual periods were available for the calculations. Rectangles with dotted edges show those bins where zero was within the interquartile range of the values in the bin, whereas continuous lines delineate such bins where zero was outside the interquartile range.

In order to accurately evaluate whether the past soil drought has an impact on the current ecosystem response to SSMI, the SAMI difference between the onset and termination needs to be accounted for. We tried to accomplish this with to the following method: first, we calculated ΔGPP bin-averages in SSMI and SAMI bins without considering the drought period lengths, akin to Fig. 6d in the article. Second, we sampled values from these ΔGPP bin-averages for each onset and termination of each drought period from their corresponding bins and calculated their difference. This gave us estimates for ΔGPP differences that were independent from the past drought period lengths, i.e. they reflect only the instantaneous ΔGPP SSMI-SAMI dependence. Finally, we subtracted these differences from the differences calculated separately for each drought period that were already described above. Figure S13d shows this adjusted difference where the SAMI dependence is accounted for. These adjusted values vary close to zero, suggesting that the differences shown in Fig. S13c were largely due to different SAMI conditions at the onset and termination of the studied periods and were not related to the past drought period length. Finally, these results suggest that the past states (i.e. length of the drought period before the current time step) were not strongly altering the ecosystem responses to the current SAMI-SSMI state in the time series utilized here. While Fig. S13 shows this only for FI-Hyy GPP, the results are qualitatively similar also at the other sites, and also for NEP and EF, however with less clear signals due to shorter flux time series available at the other sites. Furthermore, these results support our data analysis approach where we assess ecosystem drought responses to be instantaneous and relate to the current state of the system.

Effect of the time period used when estimating the drought impacts on annual scale

For testing the robustness of the annual drought impact estimates, they were estimated with the same procedure as described in the article but using flux data only from the peak growing season months (June-August). If the results are similar even when limiting the analysis to these midsummer months, then it suggests that the annual estimates reported in the article were not strongly altered by the early or late growing season conditions. Figures S14 and S15 show annual estimates of drought effects on GPP and NEP, respectively, when using data from the whole growing season as in the manuscript (blue violins) and only the three midsummer months (orange violins). Medians from the two distributions for each year and site were compared by bootstrapping the data and calculating the statistical significance of the difference between the medians.

During some of the study years at all the sites the two approaches produced different medians, i.e. the estimates of drought effects on annual scale depend on which time periods were used in the estimation procedure (the whole growing season vs June-Aug), however the distributions were largely overlapping. Note that when constraining the analysis only to the midsummer months, the effects of early and late growing season droughts on annual fluxes are completely neglected which likely causes an underestimation and also partly explains the found differences. Nevertheless, the general pattern stayed the same even when focusing only on June-Aug periods, meaning for instance that for FI-Hyy 1999, 2006 and 2018 stand out as the most severe drought years and at SE-Htm during 2018 annual GPP was estimated to be strongly decreased by droughts. For instance for FI-Hyy and SE-Htm the 2018 drought impact on annual GPP was approximated to be -120 g(C) m^{-2} a^{-1} (-140…-100 g(C) m^{-2} a^{-1}, 95 % confidence interval) and -230 g(C) m^{-2} a^{-1} (-280…-170 g(C) m^{-2} a^{-1}) when estimating only from the midsummer months and -140 g(C) m^{-2} a^{-1} (-170…-120 g(C) m^{-2} a^{-1}) and -240 g(C) m^{-2} a^{-1} (-290…-180 g(C) m^{-2} a^{-1}) when estimated from the whole growing season (as in the manuscript), respectively. For reference, the annual GPP was on average 1100 g(C) m^{-2} a^{-1} and 2220 g(C) m^{-2} a^{-1} at FI-Hyy and SE-Htm, respectively, suggesting that the adjustments in annual drought influence estimates were on the order of one percent of the typical annual GPP. For the annual NEP the drought influence estimates were -70 g(C) m^{-2} a^{-1} (-80…-50 g(C) m^{-2} a^{-1}) and -20 g(C) m^{-2} a^{-1} (-80…20 g(C) m^{-2} a^{-1}) when estimating from the midsummer months and -90 (-120…-70 g(C) m^{-2} a^{-1}) and -80 g(C) m^{-2} a^{-1} (-150…-20 g(C) m^{-2} a^{-1}) when estimating from the whole growing season, respectively. For reference, the mean annual NEP were 230 g(C) m^{-2} a^{-1} and 190 g(C) m^{-2} a^{-1} at FI-Hyy and SE-Htm, respectively.

Interestingly, the estimated decrease of annual GPP and NEP for the year 2018 due to drought decreased at several sites when considering data only from June-Aug (Figs. S14 and S15). During the year 2018 atmospheric droughts (high VPD, low SAMI) started already early May at several sites and soil droughts (low soil moisture, low SSMI) lasted until September and these early and late growing season droughts were not considered when focusing only on the midsummer months. This likely explains the differences between the estimates for the year 2018 reported above. In conclusion, these analyses suggest that the daily-to-annual scaling procedure used to estimate the drought effects on annual scale is only mildly sensitive to the period used in the scaling (the whole growing season vs June-Aug).

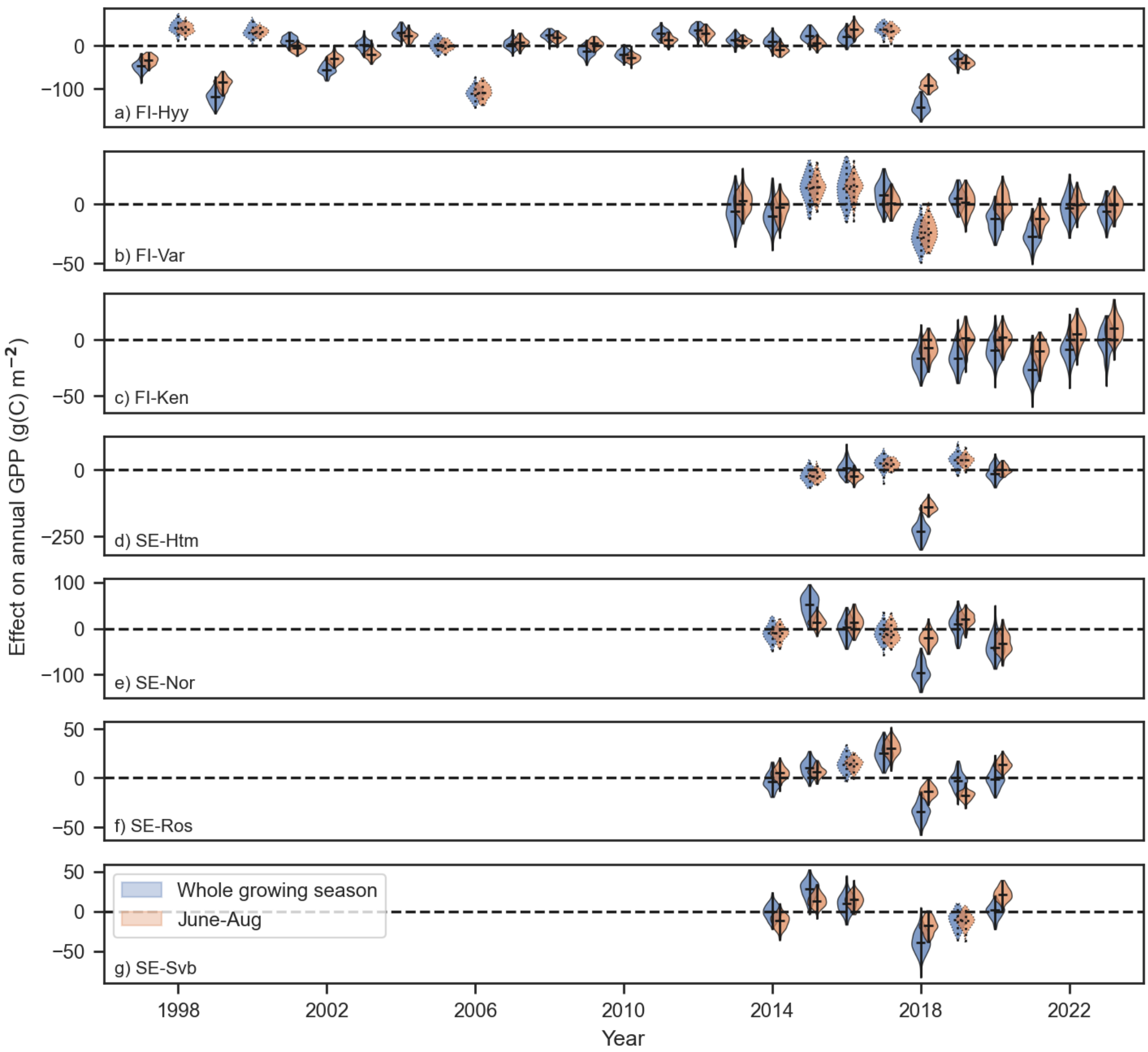

Figure S14. Effect of droughts on annual GPP at the study sites. Negative values at a given year indicate decrease of annual GPP due to stronger-than-average droughts. Horizontal lines show distribution medians. Note the y-axis scale differs between the subplots. The blue violins are the same as shown in the manuscript Fig. 7, whereas orange violins show estimates obtained using data only from June, July and August. The medians and edges of the violins are plotted with dotted lines if the two distribution medians were not significantly different from each other (p>0.05).

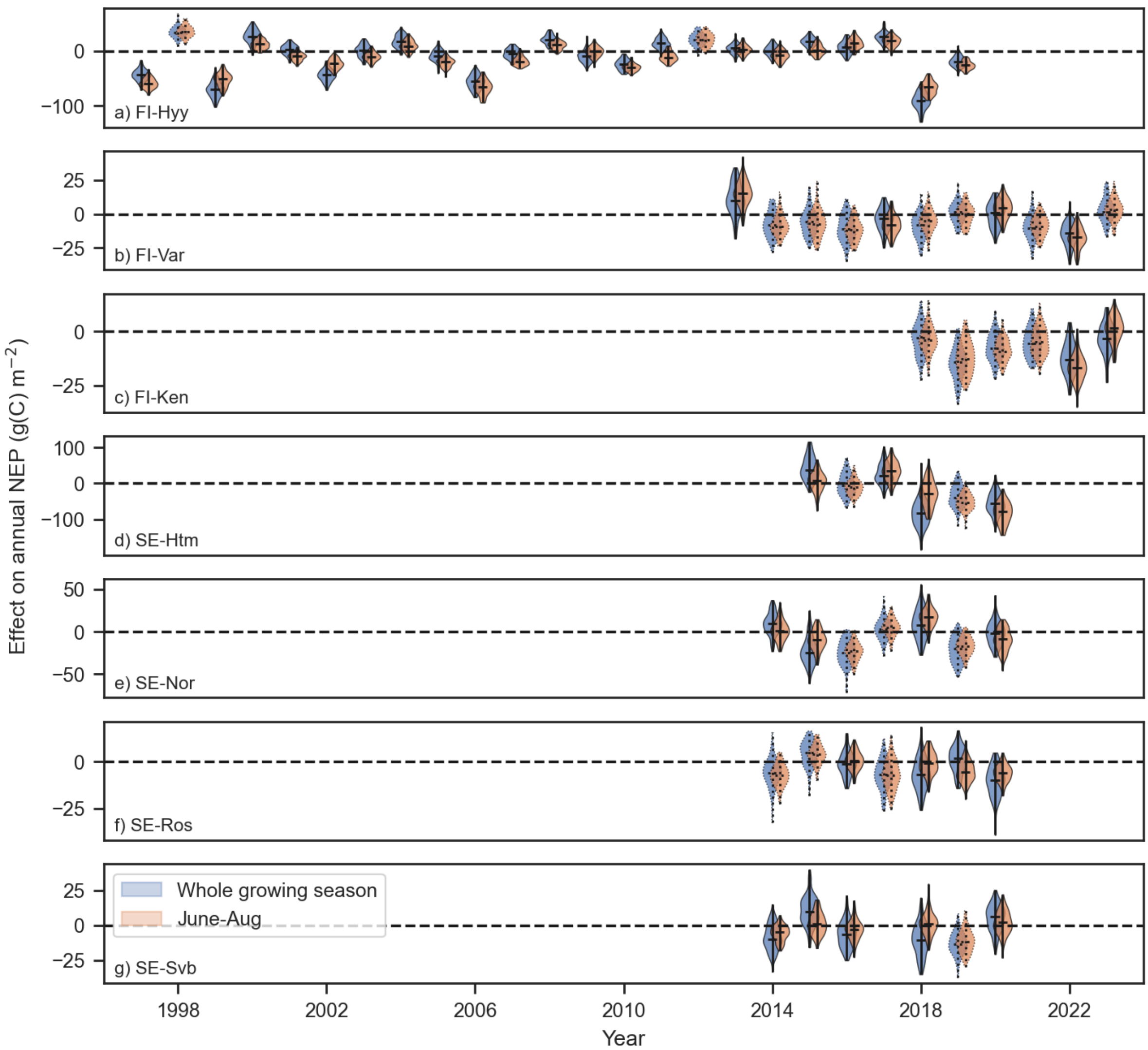

Figure S15. Same as Fig. S14 but for NEP. Note that the y-axis scales vary between the subplots and between Fig. S14 and this figure.

References

Aalto, J., Aalto, P., Keronen, P., Kolari, P., Rantala, P., Taipale, R., Kajos, M., Patokoski, J., Rinne, J., Ruuskanen, T., Leskinen, M., Laakso, H., Levula, J., Pohja, T., Siivola, E., Kulmala, M., and Ylivinkka, I.: SMEAR II Hyytiälä forest meteorology, greenhouse gases, air quality and soil, https://doi.org/10.23729/62f7ad2c-7fe0-4f66-b0a4-8d57c80524ec, 2022.

Brunner, I., Herzog, C., Dawes, M. A., Arend, M., and Sperisen, C.: How tree roots respond to drought, Frontiers in Plant Science, Volume 6-2015, https://doi.org/10.3389/fpls.2015.00547, 2015.

Choat, B., Jansen, S., Brodribb, T. J., Cochard, H., Delzon, S., Bhaskar, R., Bucci, S. J., Feild, T. S., Gleason, S. M., Hacke, U. G., Jacobsen, A. L., Lens, F., Maherali, H., Martínez-Vilalta, J., Mayr, S., Mencuccini, M., Mitchell, P. J., Nardini, A., Pittermann, J., Pratt, R. B., Sperry, J. S., Westoby, M., Wright, I. J., and Zanne, A. E.: Global convergence in the vulnerability of forests to drought, Nature, 491, 752–755, https://doi.org/10.1038/nature11688, 2012.

Novick, K. A., Ficklin, D. L., Grossiord, C., Konings, A. G., Martínez-Vilalta, J., Sadok, W., Trugman, A. T., Williams, A. P., Wright, A. J., Abatzoglou, J. T., Dannenberg, M. P., Gentine, P., Guan, K., Johnston, M. R., Lowman, L. E. L., Moore, D. J. P., and McDowell, N. G.: The impacts of rising vapour pressure deficit in natural and managed ecosystems, Plant, Cell & Environment, 47, 3561–3589, https://doi.org/10.1111/pce.14846, 2024.

Pohl, F., Rakovec, O., Rebmann, C., Hildebrandt, A., Boeing, F., Hermanns, F., Attinger, S., Samaniego, L., and Kumar, R.: Long-term daily hydrometeorological drought indices, soil moisture, and evapotranspiration for ICOS sites, Scientific Data, 10, 281, https://doi.org/10.1038/s41597-023-02192-1, 2023.
